# The *Staphylococcus aureus* Two-Component System AgrAC Displays Four Distinct Genomic Arrangements That Delineate Genomic Virulence Factor Signatures

**DOI:** 10.3389/fmicb.2018.01082

**Published:** 2018-05-25

**Authors:** Kumari S. Choudhary, Nathan Mih, Jonathan Monk, Erol Kavvas, James T. Yurkovich, George Sakoulas, Bernhard O. Palsson

**Affiliations:** ^1^Systems Biology Research Group, Department of Bioengineering, University of California, San Diego, San Diego, CA, United States; ^2^Bioinformatics and Systems Biology Program, University of California, San Diego, San Diego, CA, United States; ^3^Department of Pediatrics, University of California, San Diego, San Diego, CA, United States

**Keywords:** *Staphylococcus*, AgrAC, *agr* types, genomic arrangements, virulence, bioinformatics, microbiology

## Abstract

Two-component systems (TCSs) consist of a histidine kinase and a response regulator. Here, we evaluated the conservation of the AgrAC TCS among 149 completely sequenced *Staphylococcus aureus* strains. It is composed of four genes: *agrBDCA*. We found that: (i) AgrAC system (*agr*) was found in all but one of the 149 strains, (ii) the *agr* positive strains were further classified into four *agr* types based on AgrD protein sequences, (iii) the four *agr* types not only specified the chromosomal arrangement of the *agr* genes but also the sequence divergence of AgrC histidine kinase protein, which confers signal specificity, (iv) the sequence divergence was reflected in distinct structural properties especially in the transmembrane region and second extracellular binding domain, and (v) there was a strong correlation between the *agr* type and the virulence genomic profile of the organism. Taken together, these results demonstrate that bioinformatic analysis of the *agr* locus leads to a classification system that correlates with the presence of virulence factors and protein structural properties.

## Introduction

*Staphylococcus aureus* is a gram positive human pathogen that has evolved considerable antimicrobial resistance during the clinical antibiotic era. It can cause a wide spectrum of infection types and severity, including soft tissue infection, bloodstream infections, pneumonia, osteomyelitis, and nosocomial device related infections (Lowy, 1998; Yarwood and Schlievert, 2003; Tong et al., 2015). The wide range of pathogenicity of *S. aureus* can be attributed to its ability to produce various secreted virulence factors, such as enterotoxin, hemolysin (*hla*), serine proteases (SspA), and TSST-1 (toxic shock syndrome toxin-1), as well as those mediating cell adhesion and host evasion (Nizet, 2007). Most of the virulence factors are regulated by a two-component system (TCS), AgrAC. AgrAC TCS is encoded by the *agr* locus and acts as a quorum sensing (QS) system in *S. aureus*. This QS mechanism modulates gene expression based on population density in response to environmental stimuli.

The *agr* locus of *S. aureus* contains four genes: *agrB, agrD, agrC*, and *agrA*. *agrD* encodes for pre-peptide signal AgrD, which is modified to mature QS autoinducing peptide (AIP) and secreted to the extracellular space by the protein AgrB. *agrC* and *agrA* encode for histidine kinase and its cognate response regulator, respectively, and form the TCS. The *agr* operon can classify *S. aureus* strains into four variants or *agr* types (Novick et al., 1995; Jarraud et al., 2000; Dufour et al., 2002). These four variants (*agr* types) produce different AIPs that are characterized by different motifs of varying length. The AIP of one group has been identified to cross-inhibit the *agr* expression in the other groups (Jarraud et al., 2000; Shopsin et al., 2003; Geisinger et al., 2009). While much is known about the divergence of the *agr* variants (Dufour et al., 2002), most studies have focused on host proclivity (e.g., humans, bovine, etc.) (Jarraud et al., 2002; Lim et al., 2012; Wan et al., 2013; Schmidt et al., 2017) or on geographical location (Zaraket et al., 2007; Xie et al., 2011; Liu et al., 2012).

In this study, we predicted the *agr* type of all the completely sequenced *S. aureus* strains, irrespective of the host type or geographical location, and analyzed the divergence amongst the four *agr* types. We also proposed the evolutionary implications of these divergences.

## Materials and Methods

### *S. aureus* Genomic Data

The genomic sequences of 149 completely sequenced *S. aureus* strains were downloaded from the PATRIC database (sequenced until December 2016) (Gillespie et al., 2011) and re-annotated them with the Prokka v1.12 (Seemann, 2014) annotation tool to identify protein coding genes. The re-annotation was performed to standardize annotations across all genomes. Draft genomes were excluded from the analysis because of their incompleteness.

### *In Silico* Prediction of the *agr* Locus

The protein concatenated FASTA files of all the acquired 149 strains of *S. aureus* obtained from Prokka were searched for the presence of the domains of histidine kinases and response regulators using HMMsearch from HMMER package (Eddy, 1998). Hidden Markov Model (HMM) profiles of the histidine kinases and response regulators from Pfam database were utilized to scan the protein sequences. An *e*-value of 0.01 and score ≥ 0.25 was taken as a threshold to filter the hits from HMMsearch. For identifying the *agr* operon, neighborhood genes were scanned for the presence of *agrB* and *agrD* genes from the protein feature table obtained from Prokka.

### Classification of Strains Based on AgrD and Detection of Chromosomal Arrangement in *agr* Types

Based on the four types of AIPs in *S. aureus*, we classified *S. aureus* strains into four *agr* types. Protein sequences of AgrD for all the *S. aureus* strains were scanned for the presence of the conserved motifs that are present in each AIP by a custom python script. The corresponding strain was accordingly grouped into *agr* types. Further, the differences in the chromosomal arrangement of genes in the *agr* locus for each *agr* type were investigated. The chromosomal arrangement was identified in the form of “gene context,” defined by conserved intergenic distances between the genes in the *agr* locus.

### AgrC Sequence Variations Among the *agr* Types

To analyze sequence variation in AgrC across types, we utilized the ssbio package to inspect the biochemical properties of all sequences as well as mutations when aligned to a reference sequence (Mih et al., 2018). All strain sequences were loaded and those of non-standard length (∼430 residues) were ignored for this analysis. For the principal component analysis (PCA), we calculated general biochemical properties of the full sequences using the Biopython ProtParam and EMBOSS pepstats tools (Rice et al., 2000; Cock et al., 2009). These gave general descriptors such as the percentage of polar, non-polar, aromatic, small amino acids, etc. We used these descriptors to create a feature matrix which was then normalized with the Python scikit-learn package (Pedregosa et al., 2011). This feature matrix was then used for PCA, and the largest contributors to the first three principal components were then analyzed.

We conducted pairwise sequence alignments using the EMBOSS needle tool with default parameters (Rice et al., 2000) of all AgrC types to the reference AgrC sequence, set to the available crystallized protein of the cytoplasmic domain (UniProt: A0A0H2WWL2, PDB: 4BXI) (Srivastava et al., 2014). The reference sequence is a type I AgrC protein of *S. aureus* strain COL. We then analyzed the sequence diversity among types with respect to the reference sequence in terms of amino acid variations at each residue. Variations were scored by the Grantham score, which is a simple measure of biochemical and biophysical property differences of amino acids (Grantham, 1974). These scores of variations for each AgrC type, along with the frequencies of variations observed in each strain types, were plotted on the six transmembrane model of AgrC-I (Lina et al., 1998), using the Protter web service (Omasits et al., 2014). Variation within the cytoplasmic domain was additionally mapped to a homology model from SWISS-MODEL (template PDB: 4JAU) as well as the crystal structure (PDB: 4BXI). Stability changes incurred from these mutations were predicted using FoldX and the included BuildModel function (Guerois et al., 2002; Schymkowitz et al., 2005).

### Virulence Genes Prediction and Decision Tree

We downloaded experimentally studied virulence genes in *S. aureus* from virulence factor database (VFDB) (Chen et al., 2005) and did a BLAST search on the protein FASTA sequences of all the studied *S. aureus* strains (Altschul et al., 1990) to predict potential virulence genes in *S. aureus* strains. A threshold of 85% identity and *e*-value 0.001 was set to only identity significant hits. A decision tree was constructed using rpart package in R based on presence/absence of virulence genes in strains (Therneau et al., 2017).

### Classification of *agr* Types Based on Virulence Genes

We built random forest predictive model using virulence factors as features to classify samples by *agr* type using the “randomForest” package in R (Liaw et al., 2015). Random forest classifier was built by growing 5,000 trees. For detecting important virulence genes, we used variable importance measure in “randomForest” algorithm.

## Results

The aim of this study was to compare the divergence of the four *agr* types in the strains of *S. aureus*. Therefore, we first predicted the *agr* types of the available completely sequenced strains and then subsequently set out to determine the *agr* type specific differences in (i) the arrangement of genes in the *agr* locus, (ii) sequence variation in the histidine kinase AgrC, and (iii) the presence of different virulence genes.

### *agr* Types and MLST Typing

We analyzed 149 completely sequenced *S. aureus* strains from PATRIC database (Wattam et al., 2014) for the presence of *agr* locus. Of these, 148 strains were predicted to be positive for *agr* locus, based on the domain search of histidine kinase and response regulator proteins and querying the neighborhood genes for the presence of *agrB* and *agrD* (section “Materials and Methods”). Out of these 148 Agr positive strains, 85, 42, 16, and 4 strains were predicted as strains belonging to types I, II, III, and IV, respectively. This prediction was made by scanning the AgrD protein sequences by a custom python script for the presence of conserved motif that are present in each AIP type (Hawver et al., 2016). There was one exception to the type classification we observed. *S. aureus* strain TCH 959 did not have any conserved cysteine residue for AgrD and therefore could not be classified into any of the four types, thus, we classified it as “type_undefined.”

In addition to the *agr* typing, Multilocus Sequence Typing (MLST) is used as a tool to further delineate strains according to the sequence similarity (Robinson et al., 2005). Although a specific *agr* type has been linked to specific MLST groups (Wright et al., 2005), MLST groups within a specific *agr* type may show clonal divergence (Wright et al., 2005). Hence, we further classified strains within a *agr* type based on MLST grouping.

MLST grouping of these 148 *S. aureus* strains were analyzed from the PATRIC database (Wattam et al., 2014; Supplementary Data Sheet 1). This analysis was unable to identify MLST for all the strains in our study. Hence, a manual literature curation was conducted to assign MLST groups to these unclassified *S. aureus* strains. The names of the strains and their types (*agr* and MLST) are provided in the Supplementary Data Sheet 1. The MLST grouping which we curated through manual literature search is highlighted in red in Supplementary Data Sheet 1.

### Orientation of *agr* Genes Classifies *S. aureus* Strains Into Four Specificity Groups

**FIGURE 1:**
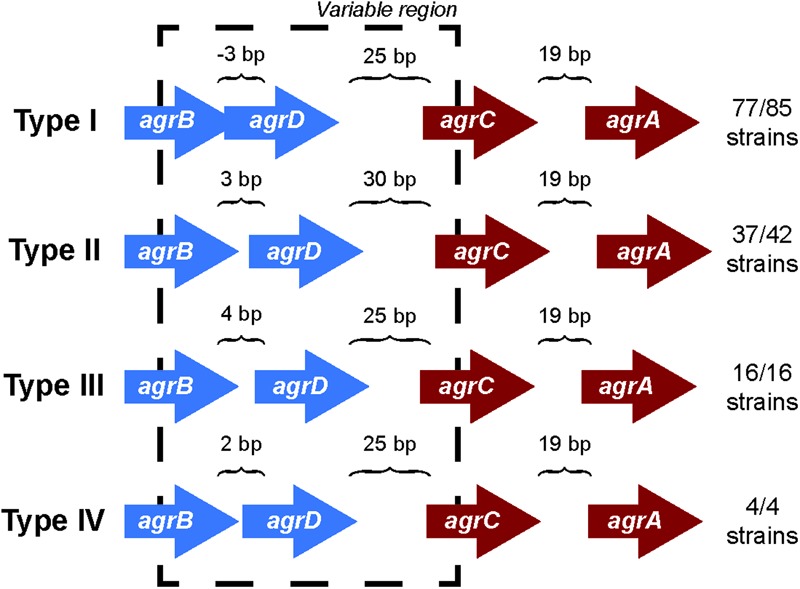
*agr* locus in different *agr* types. The intergenic distances between *agrB, agrD, agrC*, and *agrD* genes are conserved with *agr* types. The arrangement is true for 77 out of 85 strains in type I, 37 out of 42 for type II, and all the strains of type III and IV. The direction of arrows in the *agr* locus is the representation of those present in the positive strand. A similar arrangement occurs in the negative strand. The length of the arrows representing genes is not to scale. The black dotted line highlights the variable region in the *agr* locus.

Comparing the differences in the core gene arrangements within the *agr* locus would allow us to identify if the genomic features of the *agr* locus could contribute to the *agr* type specificity. To identify these differences, we analyzed the orientation of genes in the *agr* locus for each *agr* type. We found that the intergenic distances between genes in the *agr* locus differed among the four *agr* types (**Figure 1**) and were highly conserved within a specific *agr* type. For example, over 90% (77/85) of type I strains have a precise overlap of 3 bp between *agrB* and *agrD*, whereas genes are separated by 3, 4, and 2 bp in types II, III, and IV, respectively. Similarly, the intergenic distance between *agrD* and *agrC* in type II was 30 bp, while the same distance was 25 bp in the other three *agr* types. The intergenic distance between *agrC* and *agrA* was conserved and separated by 19 bp in all the four types.

However, we did observe a few non-canonical arrangements in types I (8/85) and II (5/42) which did not follow these same trends of intergenic distances indicated. Two of the type I strains (*S. aureus* strain T0131 and *S. aureus* strain HC1335) were found to contain a transposase gene between the hypothetical gene adjacent to *agrD* and *agrC* (Supplementary Figure 1). A previous study from Botelho et al. (2016) identified IS256 transposase in these two strains and suggested that this transposase truncated *agrC* into two, which inhibited the gene and subsequently the regulation of *agr* locus. While we were unable to identify any conserved motifs in the hypothetical gene between *agrD* and *agrC* to define it as a histidine kinase, we did observe five transmembrane helices. Further, to test the hypothesis whether transposase truncated *agrC* into two, we conducted a sequence alignment of the protein coding regions of the hypothetical gene between *agrD* and *agrC*, transposase, and *agrC* of these two strains with the rest of the canonical type I AgrC using Clustal Omega (Sievers and Higgins, 2014; Supplementary Figure 9). We observed that the protein coding region of the hypothetical gene between *agrD* and *agrC* and the *agrC* of *S. aureus* strain T0131 and *S. aureus* strain HC1335 aligned perfectly with the rest of the canonical type I AgrC, but there was a clear separation within the region of the transposase (these two strains are highlighted in Supplementary Figure 9). This observation may suggest that the transposase cleaved *agrC* in a manner that separated the transmembrane domain from its catalytic domain, disrupting the regulation of *agr* locus. Nevertheless, even with the insertion of transposase or a hypothetical gene, type I maintained its canonical intergenic distances of -3, 25, and 19 bp between *agrB, agrD, agrC*, and *agrA*, respectively. The type II variants seem to deviate from the canonical intergenic distances of 3, 30, and 19 bp distance between *agrB, agrD, agrC*, and *agrA*, respectively, due to the insertion of hypothetical genes in the *agr* locus (Supplementary Figure 1). These variations identify sets that may be pseudo non-functional *agr* locus, as opposed to the canonical fully functional *agr* locus.

Thus, it was clear that the gene arrangements in the *agr* locus are specific to the *agr* types which may contribute to the type specificity of the AIP recognition and allow selective advantage over the niche selection.

### AgrC Sequence Variations Among the *agr* Types

In the previous section, we identified that the gene arrangements in the *agr* locus is specific to the *agr* types, suggesting that the AgrC protein sequences may show divergence according to the type of AIP it senses (Ji et al., 1997; Dufour et al., 2002; Goerke et al., 2005; Robinson et al., 2005; Geisinger et al., 2008). Thus, we further wanted to identify specific differences in all the AgrC protein sequences among the four *agr* types. The variance in AgrC proteins amongst *agr* types was identified in two ways: (A) by investigating the overall differences in the biochemical content of the AgrC sequences among the four *agr* types and (B) by scoping out individual amino acid variations between the types.

#### Overall Differences in the Biochemical Properties of AgrC Proteins

**FIGURE 2:**
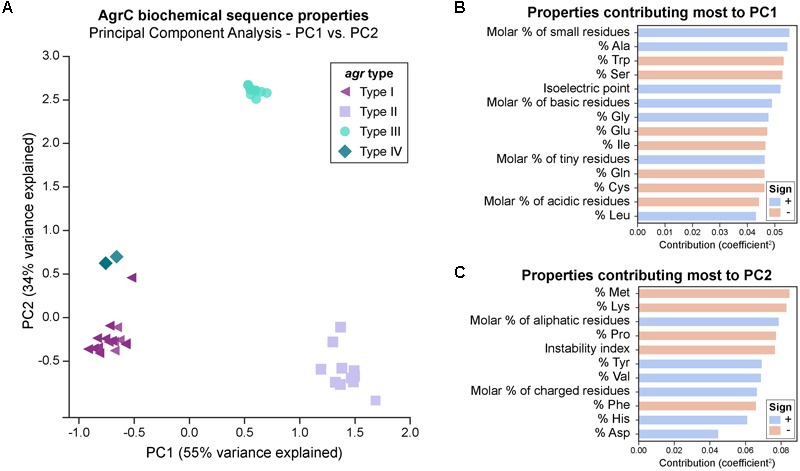
PCA of AgrC protein sequences across the *agr* types. **(A)** A total of 89% of variance is explained with the first two components. Visually, the types cluster well together. A group of six type I strains (purple triangles) cluster close to type IV strains (dark green diamonds) and are commented upon in the main text. The biochemical properties of some strains within the same *agr* type are very similar to each other and they overlap in the plot, for example: six type I strains which cluster close to type IV strains are represented by a single symbol. **(B)** Properties that contribute most to principal component 1. A positive sign (blue bar) indicates that this property increases with PC1, while a negative sign (red bar) indicates that this property decreases with PC1. Properties shown are those with a contribution score greater than 1/(total number of properties). **(C)** Properties that contribute most to principal component 2.

We first looked at characterizing differences in the basic biochemical properties of AgrC among the four *agr* types, to give us a general idea of the changes occurring at the level of the entire protein sequence. For this, we wanted to see if AgrC protein sequences clustered together as described by these biochemical properties in a PCA. The first two components (PC1-55 and PC2-34%) explained much of the variance seen. The first principal component (PC1) distinguishes between types I/IV, II, and III (**Figure 2A**). Type II proteins are enriched with a higher percentage of alanine, glycines, and leucines, with an overall greater molar percentage of small and basic residues and a higher isoelectric point (**Figure 2B**). In correspondence with these properties, type II proteins will also have a lower percentage of tryptophans, serines, glutamic acids, and isoleucines, with an overall lower molar percentage of acidic residues.

Six type I AgrC proteins (purple triangles in **Figure 2A**) four of which belong to ST59 type strains clustered near the type IV AgrC proteins (dark green diamonds), potentially suggesting that type IV may have diverged from these type I strains during evolution. Goerke et al. (2005) have also noted a subgroup of type I which was related to type IV. The third principal component (Supplementary Figure 2) explains an additional 5% of variance, and interestingly clusters these six strains alongside the type IV strains. Thus, from this component, these strains are characterized by an enrichment of polar residues, particularly histidine, with a lower molar percentage of non-polar residues. Finally, the second principal component (PC2) (**Figure 2C**) distinguished type III (cyan circles) very well from the other types and is characterized by higher molar percentage of aliphatic amino acids, charged amino acids, and valines, along with a lower percentage of methionines, lysine, prolines, phenylalanines, and a lower instability index (predicted to be more stable). We also attempted PCA on each of the domains of AgrC (extracellular or intracellular loops, transmembrane helices, or cytoplasmic domain), with the results presented in Supplementary Figure 3. Interestingly, we saw the same clustering of the types for most of the sub-domains.

#### Amino Acid Variants in AgrC Proteins

From the previous section, it was clear that the biochemical properties of the AgrC protein were specific to the *agr* type except for a few strains of type I that may form a subgroup closer to type IV. We further looked at the specific amino acid variations in the AgrC protein that may have contributed to the distinctive variations in the biochemical properties among the four *agr* types. For this analysis, we compared the protein sequences of AgrC for each *agr* type against a type I AgrC (AgrC-I) from *S. aureus* (strain COL) as a reference sequence (UniProt: A0A0H2WWL2) (Srivastava et al., 2014). This comparison was limited to AgrC proteins forming canonical gene arrangements, as those in non-canonical arrangements were mostly truncated by insertion sequences and hence amino acid variations would not be correctly identified. Furthermore, the amino acid variations observed in each *agr* type were mapped onto the predicted six transmembrane domain model (Lina et al., 1998) of the reference AgrC-I sequence A0A0H2WWL2 and were classified using Grantham scores to rate their changes on a scale of “conservative” to “radical” (see section “Materials and Methods”).

**FIGURE 3:**
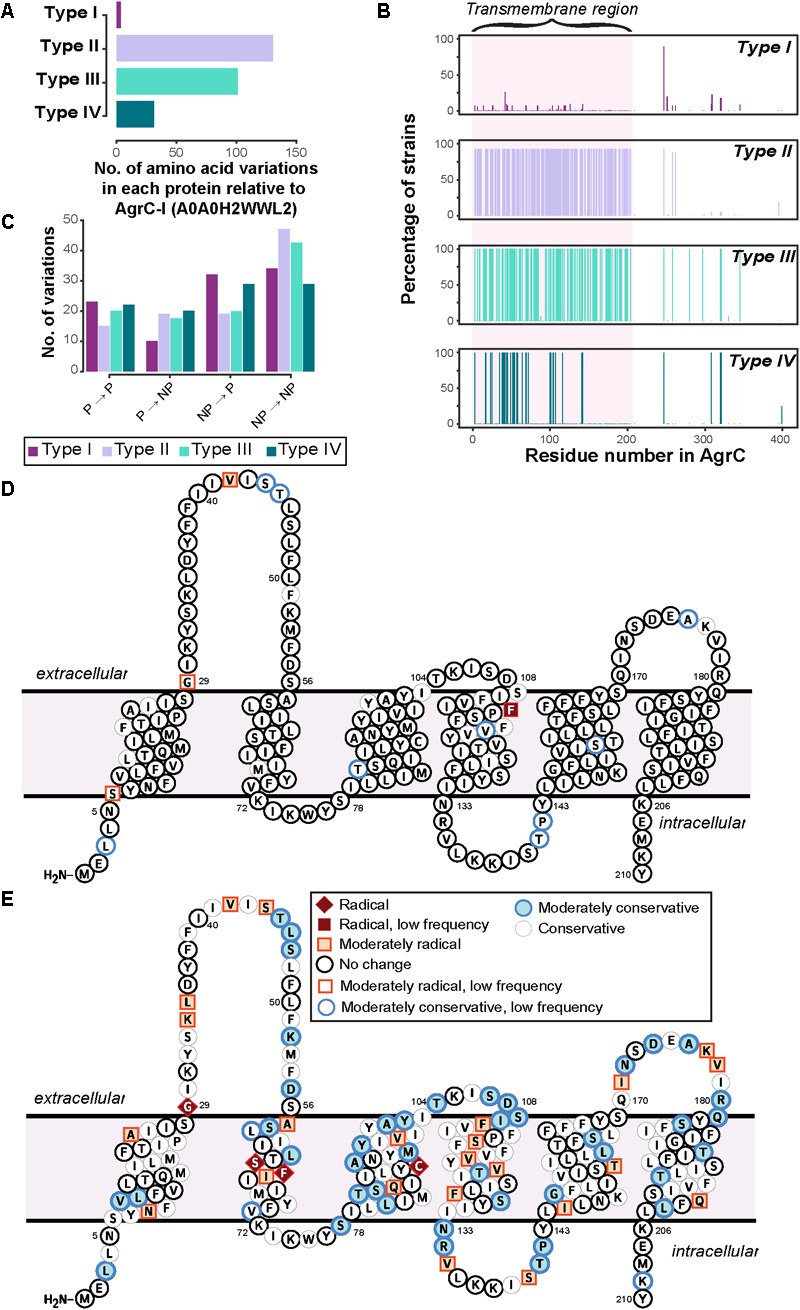
Sequence divergence in AgrC of four *agr* types. **(A)** The number of amino acid variations in each protein of *agr* types. Type I shows the least variation, as the reference strain used for the analysis is of type I. AgrC sequence of type II and type III have the highest number of amino acid variations. **(B)** The percent of strains having a variation in that residue number. Around 92 and 100% of strains of type II and type III, respectively, have variations mostly in the first 200 residues of AgrC protein. **(C)** Properties of amino acid variations in each type show that the majority of variations are between non-polar amino acids in type II and type III, meaning there is a much higher divergence in the transmembrane region. Abbreviations: NP, non-polar; P, Polar. **(D,E)**: specific amino acid divergence between type I and type II. The predicted transmembrane topology of AgrC-I reference sequence from CCTOP with highlighted amino acid residues that tend to diverge in **(D)** type I strains and **(E)** type II strains, from this study. The color coding is per biochemical and biophysical property of the amino acid residue mutated. Radical, moderate, and conservative is the nomenclature given in the order of amino acid divergence. Radical is a vast difference between amino acid properties and could change the properties of the protein. Conservative is an amino acid substitution which can be tolerated by the protein. Moderate is a biochemically moderate change in the amino acid substitution. Low frequency: amino acid variations that occurred only in very few strains.

Only a few strains of type I deviated from the reference AgrC-I sequence and, moreover, these variations were mostly conservative in nature, demonstrating that the change in the amino acids within the type I would hypothetically not affect the AgrC structure (**Figure 3** and Supplementary Figure 4). The variations observed within type I strains were seen to be dictated by the MLST typing of the strains (Supplementary Data Sheet 2), e.g., the mutation L3T occurred in six strains of type I, four of which belong to the ST59 group. A few notable changes included a mutation of V42G which appeared in 22 strains of type I (ST7, ST72, ST398, ST25, ST464) in the first extracellular loop, and F114S which appeared in only two related strains (ST25) near the predicted second extracellular loop (**Figure 3D** and Supplementary Figure 4). The AgrC sequences for types II and III strains were found to be the most divergent from the reference AgrC-I (**Figure 3A**). The amino acid variations were mainly concentrated on the first 200 residues, which comprise the N-terminal transmembrane region responsible for sensing AIPs (**Figure 3B**). We determined that in types II and III, the majority of amino acid variations were from non-polar to non-polar residues (**Figure 3C**), likely to preserve the helical transmembrane structure as they were mostly found in these regions. Some of the amino acid variations observed in type II were classified as radical changes (**Figure 3E** and Supplementary Figure 5). Specifically, a few biochemically divergent residues (e.g., G29F, S64F, F65S, C91L, marked in red in **Figure 3E**) were observed, all of which may rearrange the three-dimensional orientation of the transmembrane helices and consequently their helix–helix contacts in type II strains. In type III strains, none of variations were classified as radical changes (Supplementary Figure 6). Type IV showed very little divergence compared to type I (Supplementary Figure 7).

Finally, we inspected variations which occurred together (referred to as co-occurring mutations) in the cytoplasmic domain, of which the CA domain has been crystallized (Srivastava et al., 2014) to understand the stability of the AgrC proteins for each type. The mutation of P247 to a threonine corresponds to a phosphotransfer specificity residue (A268) found in a previous study of a similar TCS (Podgornaia et al., 2013) and does not seem to be a type specific mutation itself. This mutation was found to commonly co-occur with additional mutations at S320 and S321 which are located distal to the ATP-binding domain (Supplementary Figure 8). However, we were able to observe various combinations of co-occurring variations that were type specific, e.g., a set of seven type I AgrC proteins had the co-occurring mutations of P247T, Y251F, S320T, S321R, and T345S. The downstream effects of these mutations remain difficult to elucidate even with experimental data (Capra et al., 2010; Podgornaia et al., 2013) but likely have impacts on both the specificity and strength of binding to cognate receptors. We inspected the impact of these co-occurring mutations on the stability of the AgrC protein by using a homology model of the dimeric cytoplasmic domain, and each set of the co-occurring mutation was predicted to be significantly destabilizing (Supplementary Table 1). This destabilization suggests that these mutations may increase the propensity of the kinase to seek out its binding partners (ATP or a response regulator), then induce additional phosphotransfer reactions and lower response times to environmental stimuli. Another potential consequence would be an increase in the likelihood of non-cognate receptors to bind (Studer et al., 2013; Srivastava et al., 2014).

### Comparison of Presence of Virulence Genes in the Four Specificity *agr* Types

To further determine whether there is a correlation between the *agr* types and the presence/absence of the virulence factors, we evaluated the presence of virulence genes in all the 148 *agr* positive strains.

**FIGURE 4:**
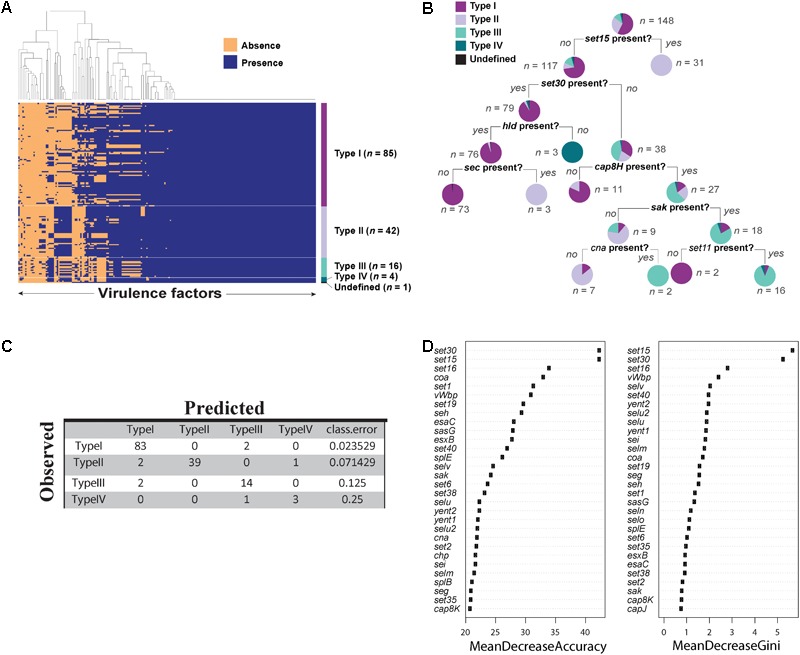
Virulence factors in *Staphylococcus aureus* strains. **(A)** Heat map showing presence and absence of virulence genes in each strain of *S. aureus*. Red depicts absence and green depicts the presence of a virulence genes. White line demarcates the heatmap into *agr* types. **(B)** Classification tree reveals differential regulation of various virulence genes in *S. aureus* dictated by *agr* types. **(C)** Accuracy of the random forest model in predicting *agr* types. **(D)** The variable importance computed by random forest algorithm.

We predicted 216 virulence genes in total to be present in the 148 *agr* positive strains. Of those predicted, 104 were present in all 148 strains (**Figure 4A**). Some of the conserved virulence genes were capsular polysaccharide genes (*cap5A-G* genes), aureolysin, autolysin, gamma, and beta hemolysin (*hlg, hlb*), genes encoding iron regulated proteins (*isd* genes), and staphylococcal protein A (*spa*). *S. aureus* strain SA564 was predicted to have the highest number of virulence genes (189 out of 216) and a mecA negative strain of *S. aureus* strain SA17_S6 was found to have the least number of virulence genes (131 out of 216) (Supplementary Data Sheet 3). Although most of the virulence genes seemed to be conserved in all the strains of *S. aureus* (**Figure 4A**), a classification tree of presence and absence of virulence genes suggested a few differences among the types (**Figure 4B**). For example, strains in which *set15* exotoxin is absent and *set30* exotoxin and *hld* are present may be characterized as type I strains. Similarly, type III can be characterized by the absence of *set15, set30*, and the presence of *cap8H*.

Next, we built a predictive model using random forest classifier to classify *agr* type based on the virulence phenotype. Random forest is a machine learning technique which builds an ensemble of classification trees and then takes the average of all the predictions to determine the class. The model showed an accuracy of 94.5% with an error rate of 5.44% in predicting the *agr* types. Of 85 type I strains, 97.6% were correctly predicted. Similarly, 92.8% of type II, 87.5% of type III, and 75% of type IV were correctly predicted by this model (**Figure 4C**). The high accuracy of the model to correctly predict the *agr* type of the strains based on virulence genes demonstrates that virulence genes can be used to discriminate *agr* types. Similar to the decision tree shown in **Figure 4B**, we identified virulence genes that are important for accurate prediction of the *agr* types (**Figure 4D**). Random forest algorithm allows two types of importance measurements: “mean decrease in accuracy” and “mean decrease in gini coefficient.” The “mean decrease in accuracy” measures how the accuracy of the model decreases if a variable is dropped. The higher the decrease in accuracy due to exclusion of a variable, the more important that variable is considered. The “mean decrease in gini coefficient” measures the contribution of the variable toward the homogeneity of the nodes in the random forest tree. We utilized the variable importance measure in random forest algorithm to identify important virulence genes.

With these results it was evident that the presence or absence of virulence factors are dependent on the *agr* type of the *S. aureus* strains.

## Discussion

In this study, we analyzed the divergence among the *agr* types of *S. aureus* with respect to the genomic features and the virulence capacity and found that the genomic features of the *agr* locus and the virulence capacity of the *S. aureus* are strongly correlated to the *agr* types.

We identified that the gene arrangement of the *agr* locus is specific to the particular *agr* types. However, we also identified some strains of type I and type II which may have incorporated a “self-destruct” mechanism of the *agr* inactivation by incorporating transposase or additional hypothetical genes in the *agr* locus. Inactivation of *agr* appears to confer a fitness advantage under the conditions of antibiotic selection, *in vivo* environment, and biofilm formation (Paulander et al., 2012; Tan et al., 2015; Botelho et al., 2016). Loss of *agr* function has been associated with resistance to host cationic antimicrobial peptides, tolerance to vancomycin, and development of intermediate resistance to vancomycin, a phenotype that is associated with treatment failure in bloodstream infections (Sakoulas et al., 2002, 2003, 2005; Mwangi et al., 2007; Tsuji et al., 2007; Fischer et al., 2011). Therefore, the subsequent study of *agr* function from isolates derived from healthcare settings as compared to those from the community, as well as *agr* function with respect to site of infection, may shed further insights on the role of *agr* inactivation in the pathogenesis of different infections.

We also identified the specific biochemical properties and the amino acid variations that distinguished AgrC proteins among the four *agr* types. The biochemical properties of the overall protein as well as the sub-domains were found to be type specific, highlighting the fact that individual domains contribute to the structure-function relationship of AgrC. We also observed that the variant *agr* alleles may form within the same *agr* types depending on the MLST typing, however, this phenomenon was more evident in AgrC type I strains. Strains belonging to ST59 had different biochemical properties and amino acid variations than the canonical *agr* type I strains. We observed that ST59 strains were more related to type IV strains; however, they had not attained a full transformation to type IV strains. A similar evolutionary model was proposed by Robinson et al. (2005) where they hypothesized that amino acid variations may occur in the *agr* locus which may affect *agr* activity beyond the specificity of the four *agr* types. The amino acid variations did seem to be concentrated in the transmembrane domain of the AgrC protein, but we could also identify specific co-occurring mutations in the catalytic domain that were type specific. We predicted that these mutations can lead to protein destabilization and change the function of the protein either by increasing the catalytic activity or by decreasing the substrate specificity (Studer et al., 2013; Srivastava et al., 2014).

Finally, we predicted *agr* type specific production of virulence genes in *S. aureus* strains (**Figure 4**). This distinction may be because different *agr* type strains secrete and recognize different AIP signals and this inherent variability to sense diverse signals correlates with pathogenesis (Yarwood and Schlievert, 2003). In addition, random forest classifier enabled us to correctly predict the *agr* type with an accuracy of 94.5%. The accuracy of the model confirmed our hypothesis that the virulence capacity is indeed correlated with the *agr* type of the strain which may give them a more competitive advantage over others. It is important to note that although ST59 strains (type I strains) have diverged from the rest of the type I strains, as seen from the amino acid variations in the AgrC sequence, the divergence is not enough to differentiate the virulence capacity of these strains.

## Conclusion

Our study suggests that during evolution, *agr* typing and the gene arrangements of the *agr* locus may have evolved together, giving rise to differential virulence capacity. The AgrC sequences subsequently diverged, giving rise to variant *agr* alleles among the same *agr* types owing to environmental pressure and competitive advantage.

## Author Contributions

KC and BP designed the research. KC, NM, and JM performed the research. KC, NM, JM, EK, JY, and GS performed analyses. KC, NM, JM, EK, JY, GS, and BP wrote the manuscript. All the authors have read and approved the manuscript.

## Conflict of Interest Statement

The authors declare that the research was conducted in the absence of any commercial or financial relationships that could be construed as a potential conflict of interest.

## References

[B1] Altschul S. F. Gish W. Miller W. Myers E. W. Lipman D. J. (1990). Basic local alignment search tool. *J. Mol. Biol.* 215 403–410. 10.1016/S0022-2836(05)80360-2 2231712

[B2] Botelho A. M. N. Costa M. O. C. Beltrame C. O. Ferreira F. A. Lima N. C. B. Costa B. S. S. (2016). Complete genome sequence of the MRSA isolate HC1335 from ST239 lineage displaying a truncated AgrC histidine kinase receptor. *Genome Biol. Evol.* 8 3187–3192. 10.1093/gbe/evw225 27635055 PMC5174738

[B3] Capra E. J. Perchuk B. S. Lubin E. A. Ashenberg O. Skerker J. M. Laub M. T. (2010). Systematic dissection and trajectory-scanning mutagenesis of the molecular interface that ensures specificity of two-component signaling pathways. *PLoS Genet.* 6:e1001220. 10.1371/journal.pgen.1001220 21124821 PMC2991266

[B4] Chen L. Yang J. Yu J. Yao Z. Sun L. Shen Y. (2005). VFDB: a reference database for bacterial virulence factors. *Nucleic Acids Res.* 33 D325–D328. 10.1093/nar/gki008 15608208 PMC539962

[B5] Cock P. J. A. Antao T. Chang J. T. Chapman B. A. Cox C. J. Dalke A. (2009). Biopython: freely available Python tools for computational molecular biology and bioinformatics. *Bioinformatics* 25 1422–1423. 10.1093/bioinformatics/btp163 19304878 PMC2682512

[B6] Dufour P. Jarraud S. Vandenesch F. Greenland T. Novick R. P. Bes M. (2002). High genetic variability of the agr locus in *Staphylococcus* species. *J. Bacteriol.* 184 1180–1186. 10.1128/jb.184.4.1180-1186.2002 11807079 PMC134794

[B7] Eddy S. R. (1998). Profile hidden Markov models. *Bioinformatics* 14 755–763. 10.1093/bioinformatics/14.9.755 9918945

[B8] Fischer A. Yang S.-J. Bayer A. S. Vaezzadeh A. R. Herzig S. Stenz L. (2011). Daptomycin resistance mechanisms in clinically derived *Staphylococcus aureus* strains assessed by a combined transcriptomics and proteomics approach. *J. Antimicrob. Chemother.* 66 1696–1711. 10.1093/jac/dkr195 21622973 PMC3133485

[B9] Geisinger E. George E. A. Chen J. Muir T. W. Novick R. P. (2008). Identification of ligand specificity determinants in AgrC, the *Staphylococcus aureus* quorum-sensing receptor. *J. Biol. Chem.* 283 8930–8938. 10.1074/jbc.M710227200 18222919 PMC2276371

[B10] Geisinger E. Muir T. W. Novick R. P. (2009). agr receptor mutants reveal distinct modes of inhibition by staphylococcal autoinducing peptides. *Proc. Natl. Acad. Sci. U.S.A.* 106 1216–1221. 10.1073/pnas.0807760106 19147840 PMC2633565

[B11] Gillespie J. J. Wattam A. R. Cammer S. A. Gabbard J. L. Shukla M. P. Dalay O. (2011). PATRIC: the comprehensive bacterial bioinformatics resource with a focus on human pathogenic species. *Infect. Immun.* 79 4286–4298. 10.1128/IAI.00207-11 21896772 PMC3257917

[B12] Goerke C. Esser S. Kümmel M. Wolz C. (2005). *Staphylococcus aureus* strain designation by agr and cap polymorphism typing and delineation of agr diversification by sequence analysis. *Int. J. Med. Microbiol.* 295 67–75. 10.1016/j.ijmm.2005.01.004 15969467

[B13] Grantham R. (1974). Amino acid difference formula to help explain protein evolution. *Science* 185 862–864. 10.1126/science.185.4154.862 4843792

[B14] Guerois R. Nielsen J. E. Serrano L. (2002). Predicting changes in the stability of proteins and protein complexes: a study of more than 1000 mutations. *J. Mol. Biol.* 320 369–387. 10.1016/S0022-2836(02)00442-4 12079393

[B15] Hawver L. A. Jung S. A. Ng W.-L. (2016). Specificity and complexity in bacterial quorum-sensing systems. *FEMS Microbiol. Rev.* 40 738–752. 10.1093/femsre/fuw014 27354348 PMC5007282

[B16] Jarraud S. Lyon G. J. Figueiredo A. M. Lina G. Gérard L. Vandenesch F. (2000). Exfoliatin-producing strains define a fourth agr specificity group in *Staphylococcus aureus*. *J. Bacteriol.* 182 6517–6522. 10.1128/JB.182.22.6517-6522.2000 11053400 PMC94802

[B17] Jarraud S. Mougel C. Thioulouse J. Lina G. Meugnier H. Forey F. (2002). Relationships between *Staphylococcus aureus* genetic background, virulence factors, agr groups (alleles), and human disease. *Infect. Immun.* 70 631–641. 10.1128/IAI.70.2.631-641.2002 11796592 PMC127674

[B18] Ji G. Beavis R. Novick R. P. (1997). Bacterial interference caused by autoinducing peptide variants. *Science* 276 2027–2030. 10.1126/science.276.5321.2027 9197262

[B19] Liaw A. Wiener M. Andy Liaw M. (2015). *Random Forests for Classification and Regression Description Classification and Regression Based on a Forest of Trees Using Random Inputs.* Available at: https://www.stat.berkeley.edu/~breiman/RandomForests/

[B20] Lim K.-T. Hanifah Y. A. Yusof M. Y. M. Thong K.-L. (2012). Characterisation of the virulence factors and genetic types of methicillin susceptible *Staphylococcus aureus* from patients and healthy individuals. *Indian J. Microbiol.* 52 593–600. 10.1007/s12088-012-0286-7 24293716 PMC3516654

[B21] Lina G. Jarraud S. Ji G. Greenland T. Pedraza A. Etienne J. (1998). Transmembrane topology and histidine protein kinase activity of AgrC, the agr signal receptor in *Staphylococcus aureus*. *Mol. Microbiol.* 28 655–662. 10.1046/j.1365-2958.1998.00830.x 9632266

[B22] Liu Q. Han L. Li B. Sun J. Ni Y. (2012). Virulence characteristic and MLST-agr genetic background of high-level mupirocin-resistant, MRSA Isolates from Shanghai and Wenzhou, China. *PLoS One* 7:e37005. 10.1371/journal.pone.0037005 22623969 PMC3356393

[B23] Lowy F. D. (1998). *Staphylococcus aureus* infections. *N. Engl. J. Med.* 339 520–532. 10.1056/NEJM199808203390806 9709046

[B24] Mih N. Brunk E. Chen K. Catoiu E. Sastry A. Kavvas E. (2018). ssbio: a python framework for structural systems biology. *Bioinformatics* 10.1093/bioinformatics/bty077 [Epub ahead of print]. 29444205 PMC6658713

[B25] Mwangi M. M. Wu S. W. Zhou Y. Sieradzki K. de Lencastre H. Richardson P. (2007). Tracking the in vivo evolution of multidrug resistance in *Staphylococcus aureus* by whole-genome sequencing. *Proc. Natl. Acad. Sci. U.S.A.* 104 9451–9456. 10.1073/pnas.0609839104 17517606 PMC1890515

[B26] Nizet V. (2007). Understanding how leading bacterial pathogens subvert innate immunity to reveal novel therapeutic targets. *J. Allergy Clin. Immunol.* 120 13–22. 10.1016/j.jaci.2007.06.005 17606031

[B27] Novick R. P. Projan S. J. Kornblum J. Ross H. F. Ji G. Kreiswirth B. (1995). The agr P2 operon: an autocatalytic sensory transduction system in *Staphylococcus aureus*. *Mol. Gen. Genet* 248 446–458. 10.1007/BF02191645 7565609

[B28] Omasits U. Ahrens C. H. Müller S. Wollscheid B. (2014). Protter: interactive protein feature visualization and integration with experimental proteomic data. *Bioinformatics* 30 884–886. 10.1093/bioinformatics/btt607 24162465

[B29] Paulander W. Nissen Varming A. Bæk K. T. Haaber J. Frees D. Ingmer H. (2012). Antibiotic-mediated selection of quorum-sensing-negative *Staphylococcus aureus*. *mBio* 3:e00459-12. 10.1128/mBio.00459-12 23143800 PMC3509436

[B30] Pedregosa F. Varoquaux G. Gramfort A. Michel V. Thirion B. Grisel O. (2011). Scikit-learn: machine learning in python. *J. Mach. Learn. Res.* 12 2825–2830.

[B31] Podgornaia A. I. Casino P. Marina A. Laub M. T. (2013). Structural basis of a rationally rewired protein-protein interface critical to bacterial signaling. *Structure* 21 1636–1647. 10.1016/j.str.2013.07.005 23954504 PMC3821218

[B32] Rice P. Longden I. Bleasby A. (2000). EMBOSS: the European molecular biology open software suite. *Trends Genet.* 16 276–277. 10.1016/S0168-9525(00)02024-2 10827456

[B33] Robinson D. A. Monk A. B. Cooper J. E. Feil E. J. Enright M. C. (2005). Evolutionary genetics of the accessory gene regulator (agr) locus in *Staphylococcus aureus*. *J. Bacteriol.* 187 8312–8321. 10.1128/JB.187.24.8312-8321.2005 16321935 PMC1317016

[B34] Sakoulas G. Eliopoulos G. M. Fowler V. G. Moellering R. C. Novick R. P. Lucindo N. (2005). Reduced susceptibility of *Staphylococcus aureus* to vancomycin and platelet microbicidal protein correlates with defective autolysis and loss of accessory gene regulator (agr) function. *Antimicrob. Agents Chemother.* 49 2687–2692. 10.1128/AAC.49.7.2687-2692.2005 15980337 PMC1168700

[B35] Sakoulas G. Eliopoulos G. M. Moellering R. C. Wennersten C. Venkataraman L. Novick R. P. (2002). Accessory gene regulator (agr) locus in geographically diverse *Staphylococcus aureus* isolates with reduced susceptibility to vancomycin. *Antimicrob. Agents Chemother.* 46 1492–1502. 10.1128/AAC.46.5.1492-1502.2002 11959587 PMC127153

[B36] Sakoulas G. Eliopoulos G. M. Moellering RC Jr Novick R. P. Venkataraman L. Wennersten C. (2003). Staphylococcus aureus accessory gene regulator (agr) group II: is there a relationship to the development of intermediate-level glycopeptide resistance? *J. Infect. Dis.* 187 929–938. 10.1086/368128 12660939

[B37] Schmidt T. Kock M. M. Ehlers M. M. (2017). Molecular characterization of *Staphylococcus aureus* isolated from bovine mastitis and close human contacts in south african dairy herds: genetic diversity and inter-species host transmission. *Front. Microbiol.* 8:511. 10.3389/fmicb.2017.00511 28428772 PMC5382207

[B38] Schymkowitz J. Borg J. Stricher F. Nys R. Rousseau F. Serrano L. (2005). The FoldX web server: an online force field. *Nucleic Acids Res.* 33 W382–W388. 10.1093/nar/gki387 15980494 PMC1160148

[B39] Seemann T. (2014). Prokka: rapid prokaryotic genome annotation. *Bioinformatics* 30 2068–2069. 10.1093/bioinformatics/btu153 24642063

[B40] Shopsin B. Mathema B. Alcabes P. Said-Salim B. Lina G. Matsuka A. (2003). Prevalence of agr specificity groups among *Staphylococcus aureus* strains colonizing children and their guardians. *J. Clin. Microbiol.* 41 456–459. 10.1128/JCM.41.1.456-459.2003 12517893 PMC149583

[B41] Sievers F. Higgins D. G. (2014). “Clustal Omega, accurate alignment of very large numbers of sequences,” in *Methods in Molecular Biology* Vol. 1079 ed. Russell D. (Totowa, NJ: Humana Press). 10.1007/978-1-62703-646-7_6 24170397

[B42] Srivastava S. K. Rajasree K. Fasim A. Arakere G. Gopal B. (2014). Influence of the AgrC-AgrA complex on the response time of *Staphylococcus aureus* quorum sensing. *J. Bacteriol.* 196 2876–2888. 10.1128/JB.01530-14 24858185 PMC4135676

[B43] Studer R. A. Dessailly B. H. Orengo C. A. (2013). Residue mutations and their impact on protein structure and function: detecting beneficial and pathogenic changes. *Biochem. J.* 449 581–594. 10.1042/BJ20121221 23301657

[B44] Tan X. Qin N. Wu C. Sheng J. Yang R. Zheng B. (2015). Transcriptome analysis of the biofilm formed by methicillin-susceptible *Staphylococcus aureus*. *Sci. Rep.* 5:11997. 10.1038/srep11997 26149474 PMC4493712

[B45] Therneau T. M. Atkinson E. J. Foundation M. (2017). *An Introduction to Recursive Partitioning Using the RPART Routines.* Available at: https://cran.r-project.org/web/packages/rpart/vignettes/longintro.pdf

[B46] Tong S. Y. C. Davis J. S. Eichenberger E. Holland T. L. Fowler V. G. Jr. (2015). *Staphylococcus aureus* infections: epidemiology, pathophysiology, clinical manifestations, and management. *Clin. Microbiol. Rev.* 28 603–661. 10.1128/CMR.00134-14 26016486 PMC4451395

[B47] Tsuji B. T. Rybak M. J. Lau K. L. Sakoulas G. (2007). Evaluation of accessory gene regulator (agr) group and function in the proclivity towards vancomycin intermediate resistance in *Staphylococcus aureus*. *Antimicrob. Agents Chemother.* 51 1089–1091. 10.1128/AAC.00671-06 17158941 PMC1803123

[B48] Wan M. T. Lauderdale T. L. Chou C. C. (2013). Characteristics and virulence factors of livestock associated ST9 methicillin-resistant *Staphylococcus aureus* with a novel recombinant staphylocoagulase type. *Vet. Microbiol.* 162 779–784. 10.1016/j.vetmic.2012.10.003 23116588

[B49] Wattam A. R. Abraham D. Dalay O. Disz T. L. Driscoll T. Gabbard J. L. (2014). PATRIC, the bacterial bioinformatics database and analysis resource. *Nucleic Acids Res.* 42 D581–D591. 10.1093/nar/gkt1099 24225323 PMC3965095

[B50] Wright J. S. III Traber K. E. Corrigan R. Benson S. A. Musser J. M. Novick R. P. (2005). The agr radiation: an early event in the evolution of staphylococci. *J. Bacteriol.* 187 5585–5594. 10.1128/JB.187.16.5585-5594.2005 16077103 PMC1196086

[B51] Xie Y. He Y. Gehring A. Hu Y. Li Q. Tu S.-I. (2011). Genotypes and toxin gene profiles of *Staphylococcus aureus* clinical isolates from China. *PLoS One* 6:e28276. 10.1371/journal.pone.0028276 22194821 PMC3240617

[B52] Yarwood J. M. Schlievert P. M. (2003). Quorum sensing in Staphylococcus infections. *J. Clin. Invest.* 112 1620–1625. 10.1172/JCI20442 14660735 PMC281656

[B53] Zaraket H. Otsuka T. Saito K. Dohmae S. Takano T. Higuchi W. (2007). Molecular characterization of methicillin-resistant *Staphylococcus aureus* in hospitals in Niigata, Japan: divergence and transmission. *Microbiol. Immunol.* 51 171–176. 10.1111/j.1348-0421.2007.tb03898.x 17310084

