# Supplementary material for: The Staphylococcus aureus Two-Component System AgrAC Displays Four Distinct Genomic Arrangements That Delineate Genomic Virulence Factor Signatures
Source: Front Microbiol. 2018 May 25;9:1082. doi: 10.3389/fmicb.2018.01082 (PMC5981134; doi:10.3389/fmicb.2018.01082)
Supplement: Supplementary file 1 [file Table_1.PDF]

Supplementary Material

The *Staphylococcus aureus* Two-Component System AgrAC Displays Four Distinct Genomic Arrangements That Delineate Genomic Virulence Factor Signatures

Kumari Sonal Choudhary[1], Nathan Mih[1,2], Jonathan Monk[1], Erol Kavvas[1], James T. Yurkovich[1,2], George Sakoulas[3], Bernhard O. Palsson[1,2,3*]

[1]Systems Biology Research Group, Department of Bioengineering, University of California, San Diego, CA
[2]Bioinformatics and Systems Biology Program, University of California, San Diego
[3]Department of Pediatrics, University of California, San Diego

***Correspondence**:
Bernhard O. Palsson
palsson@eng.ucsd.edu

SUPPLEMENTARY TABLE

Table 1: Co-occurring mutations seen in the cytoplasmic domain with residues P247, S320, and S321. See Supplementary Image 8 for a visual representation of these residues and their proximity to important binding sites. An asterisk indicates that these residues are in the location of previously studied phosphotransfer specificity residues, which bind to the response regulator. The impact of these mutations on protein stability was predicted using FoldX on a homology model (PDB ID 4LAU used as the template) of the dimer form of the cytoplasmic domain. The impact in stability is presented as a ΔΔG (kcal/mol), where positive values indicate destabilizing mutants.

**DHp domain**			**CA domain**							**Appearance in *agr* type**				**Stability impact**
* *P247T*	*Y251F*	*P258T*	*I280L*	*I297L*	*N308S*	*S320T*	*S321R*	*S321H*	*T345S*	I	II	III	IV	ΔΔG
x					x	x		x		6	2		3	4.7 ± 0.7
x		x	x	x		x	x		x			14		7.6 ± 1.0
x	x					x	x		x	7				3.8 ± 0.5
x					x	x	x			2				6.6 ± 0.8
