# Supplementary material for: The Staphylococcus aureus Two-Component System AgrAC Displays Four Distinct Genomic Arrangements That Delineate Genomic Virulence Factor Signatures
Source: Front Microbiol. 2018 May 25;9:1082. doi: 10.3389/fmicb.2018.01082 (PMC5981134; doi:10.3389/fmicb.2018.01082)
Supplement: Supplementary file 2 [file Image_1.PDF]

Supplementary Material

The *Staphylococcus aureus* Two-Component System AgrAC Displays Four Distinct Genomic Arrangements That Delineate Genomic Virulence Factor Signatures

Kumari Sonal Choudhary[1], Nathan Mih[1,2], Jonathan Monk[1], Erol Kavvas[1], James T. Yurkovich[1,2], George Sakoulas[3], Bernhard O. Palsson[1,2,3*]

[1]Systems Biology Research Group, Department of Bioengineering, University of California, San Diego, CA
[2]Bioinformatics and Systems Biology Program, University of California, San Diego
[3]Department of Pediatrics, University of California, San Diego

***Correspondence**:
Bernhard O. Palsson
palsson@eng.ucsd.edu

SUPPLEMENTARY FIGURE

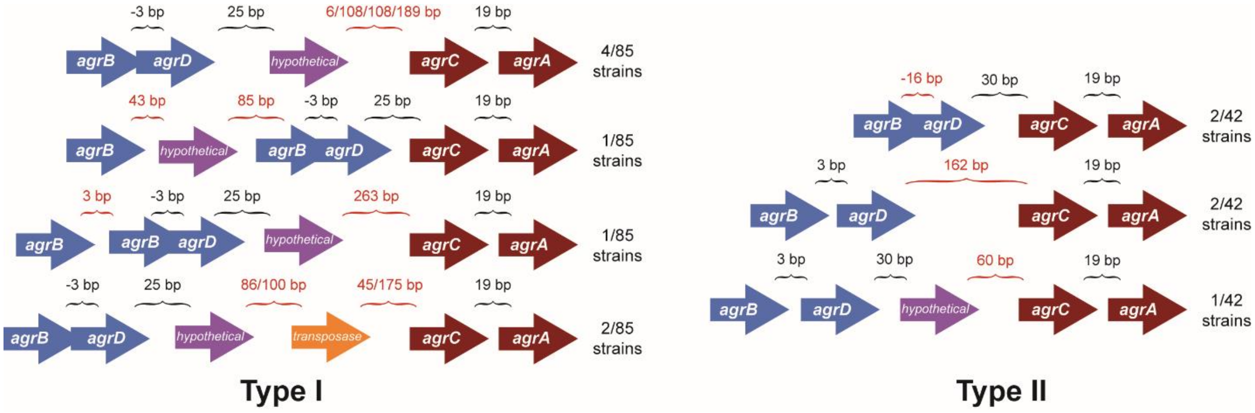

Figure 1. Variations in the canonical arrangement of *agr* locus in type I and type II strains. Intergenic distances marked in red agree with the canonical arrangement.
