# Supplementary material for: The Staphylococcus aureus Two-Component System AgrAC Displays Four Distinct Genomic Arrangements That Delineate Genomic Virulence Factor Signatures
Source: Front Microbiol. 2018 May 25;9:1082. doi: 10.3389/fmicb.2018.01082 (PMC5981134; doi:10.3389/fmicb.2018.01082)
Supplement: Supplementary file 8 [file Image_7.PDF]

*Supplementary Material*

# The *Staphylococcus aureus* Two-Component System AgrAC Displays Four Distinct Genomic Arrangements That Delineate Genomic Virulence Factor Signatures

Kumari Sonal Choudhary[1], Nathan Mih[1,2], Jonathan Monk[1], Erol Kavvas[1], James T. Yurkovich[1,2], George Sakoulas[3], Bernhard O. Palsson[1,2,3*]

[1]Systems Biology Research Group, Department of Bioengineering, University of California, San Diego, CA
[2]Bioinformatics and Systems Biology Program, University of California, San Diego
[3]Department of Pediatrics, University of California, San Diego

***Correspondence**:
Bernhard O. Palsson
palsson@eng.ucsd.edu

## SUPPLEMENTARY FIGURE

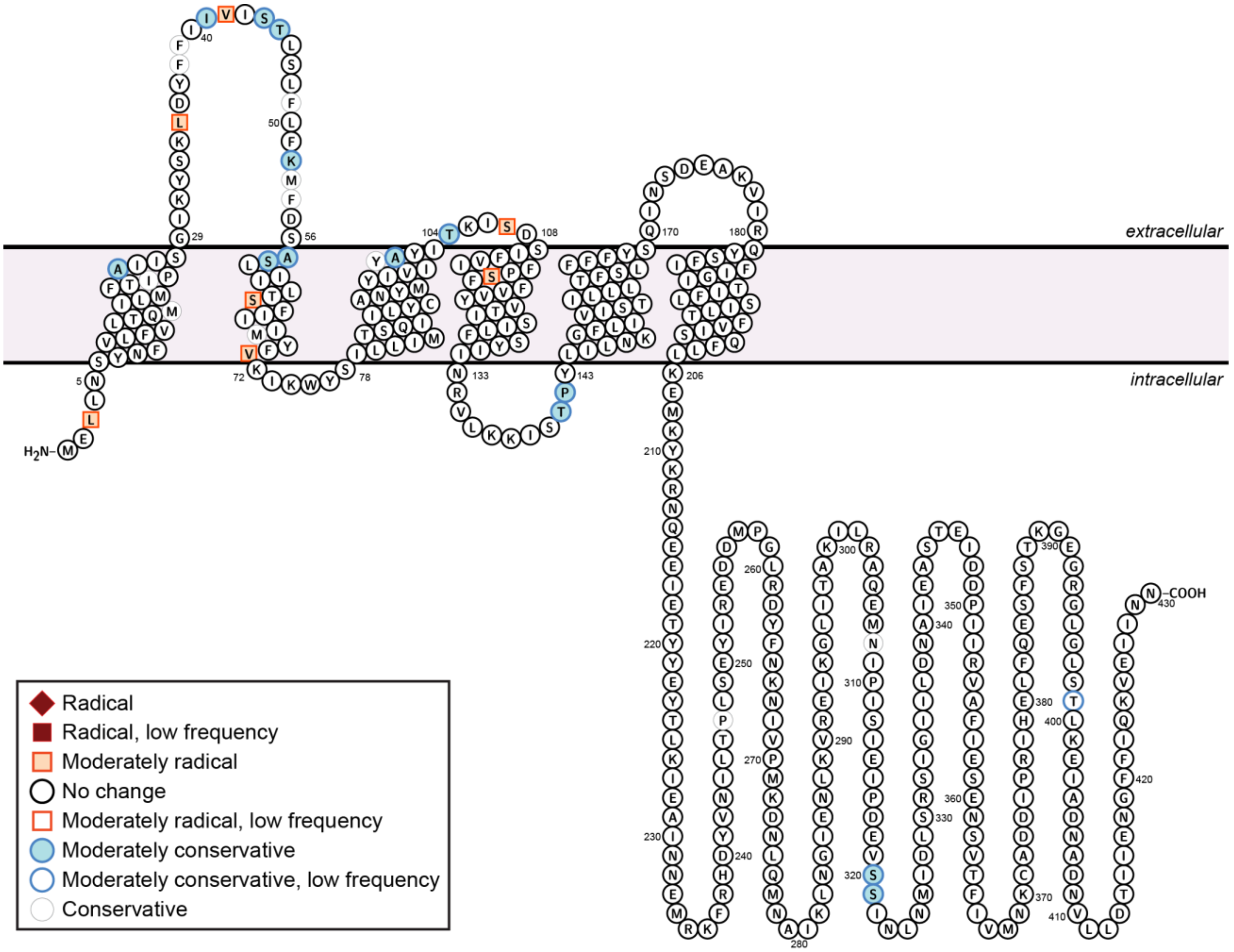


**Figure 7: Comparison of AgrC in type IV strains with AgrC-I reference sequence.** The predicted topology of AgrC-I reference sequence from CCTOP with highlighted amino acid residues that tend to diverge in type IV strains. The color coding is per biochemical and biophysical property of the amino acid residue mutated. Radical, moderate and conservative are the nomenclature given in the order of amino acid divergence with radical being vast difference between amino acid properties and could change the properties of the protein. Conservative change depicts the amino acid substitution which can be tolerated by the protein. Low frequency: amino acid variations that occurred only in very few strains.
