# Supplementary material for: The Staphylococcus aureus Two-Component System AgrAC Displays Four Distinct Genomic Arrangements That Delineate Genomic Virulence Factor Signatures
Source: Front Microbiol. 2018 May 25;9:1082. doi: 10.3389/fmicb.2018.01082 (PMC5981134; doi:10.3389/fmicb.2018.01082)
Supplement: Supplementary file 9 [file Image_8.PDF]

Supplementary Material

The *Staphylococcus aureus* Two-Component System AgrAC Displays Four Distinct Genomic Arrangements That Delineate Genomic Virulence Factor Signatures

Kumari Sonal Choudhary[1], Nathan Mih[1,2], Jonathan Monk[1], Erol Kavvas[1], James T. Yurkovich[1,2], George Sakoulas[3], Bernhard O. Palsson[1,2,3*]

[1]Systems Biology Research Group, Department of Bioengineering, University of California, San Diego, CA
[2]Bioinformatics and Systems Biology Program, University of California, San Diego
[3]Department of Pediatrics, University of California, San Diego

***Correspondence**:
Bernhard O. Palsson
palsson@eng.ucsd.edu

SUPPLEMENTARY FIGURE

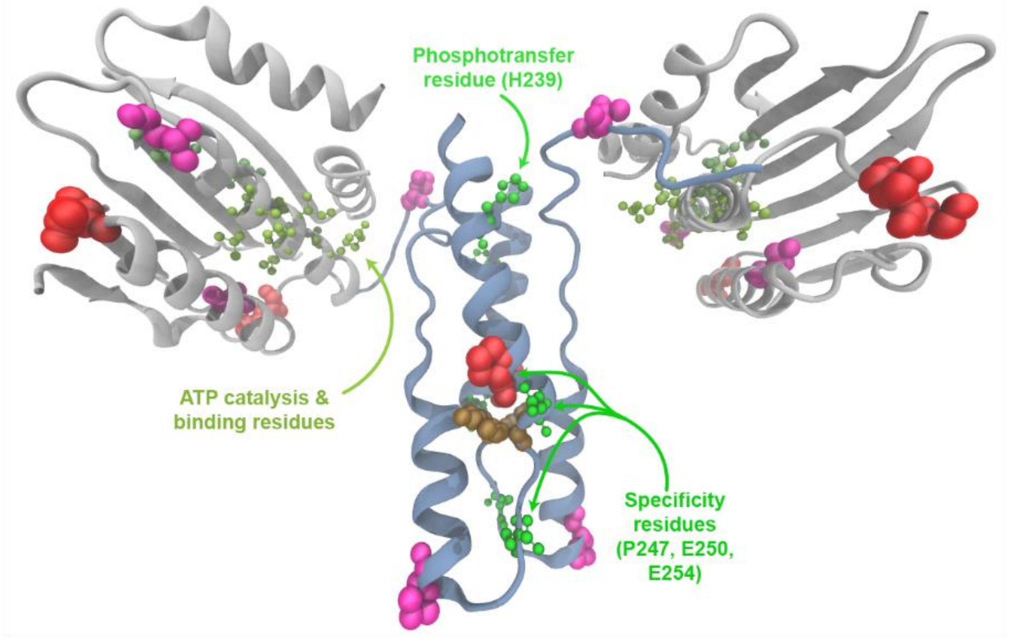

Figure 8: 3D structural representation of popular co-occurring mutations contained in the cytoplasmic domain, using a homology model from SWISS-MODEL based on PDB ID 4JAU. The CA domain (silver) is an overlay of the experimental AgrC structure (PDB ID 4BXI) over the homology model, which shows the DHp domain (blue). Residues in green are putative binding or catalytic residues, with the phosphotransfer residue (His239), response regulator specificity residues (Pro247, Glu250, Glu254), and ATP binding/hydrolysis residues highlighted. Residues in red, purple, and brown are co-occurring mutation sets that were found throughout the types, and described in Supplemental Table ST1. Note that some sets have residues that overlap each other.
