# Supplementary material for: The Staphylococcus aureus Two-Component System AgrAC Displays Four Distinct Genomic Arrangements That Delineate Genomic Virulence Factor Signatures
Source: Front Microbiol. 2018 May 25;9:1082. doi: 10.3389/fmicb.2018.01082 (PMC5981134; doi:10.3389/fmicb.2018.01082)
Supplement: Supplementary file 10 [file Image_9.PDF]

frontiers

Supplementary Material

The *Staphylococcus aureus* Two-Component System AgrAC Displays Four Distinct Genomic Arrangements That Delineate Genomic Virulence Factor Signatures

Kumari Sonal Choudhary[1], Nathan Mih[1,2], Jonathan Monk[1], Erol Kavvas[1], James T. Yurkovich[1,2], George Sakoulas[3], Bernhard O. Palsson[1,2,3*]

[1]Systems Biology Research Group, Department of Bioengineering, University of California, San Diego, CA
[2]Bioinformatics and Systems Biology Program, University of California, San Diego
[3]Department of Pediatrics, University of California, San Diego

***Correspondence**:
Bernhard O. Palsson
palsson@eng.ucsd.edu

SUPPLEMENTARY FIGURE

```
CLUSTAL O(1.2.4) multiple sequence alignment


1368166.3_typeI       METLNSYNFVLFVVTQMILMFTIPALISGIKYSKLDYFFIIVITSLSLFLYKMFDSASLI  60
1280.10759_typeI      METLNSYNFVLFVVTQMILMFTIPALISGIKYSKLDYFFIIVITSLSLFLYKMFDSASLI  60
1118959.3_typeI       METLNSYNFVLFVVTQMILMFTIPALISGIKYSKLDYFFIIVITSLSLFLYKMFDSASLI  60
685039.3_typeI        METLNSYNFVLFVVTQMILMFTIPALISGIKYSKLDYFFIIVITSLSLFLYKMFDSASLI  60
1201010.3_typeI       METLNSYNFVLFVVTQMILMFTIPALISGIKYSKLDYFFIIVITSLSLFLYKMFDSASLI  60
1194085.3_typeI       METLNSYNFVLFVVTQMILMFTIPALISGIKYSKLDYFFIIVITSLSLFLYKMFDSASLI  60
1280.3367_typeI       MELLNSYNFVLFVLTQMILMFTIPAIISGIKYSKLDYFFIIGITTLSLFLFKMFDSASLI  60
1280.5205_typeI       MELLNSYNFVLFVLTQMILMFTIPAIISGIKYSKLDYFFIIGITTLSLFLFKMFDSASLI  60
523796.5_typeI        MELLNSYNFVLFVLTQMILMFTIPAIISGIKYSKLDYFFIIGISTLSLFLFKMFDSASLI  60
1280.4850_typeI       MELLNSYNFVLFVLTQMILMFTIPAIISGIKYSKLDYFFIIGISTLSLFLFKMFDSASLI  60
1280.4852_typeI       MELLNSYNFVLFVLTQMILMFTIPAIISGIKYSKLDYFFIIGISTLSLFLFKMFDSASLI  60
1155084.3_typeI       MELLNSYNFVLFVLTQMILMFTIPAIISGIKYSKLDYFFIIGISTLSLFLFKMFDSASLI  60
46170.288_typeI       MELLNSYNFVLFVLTQMILMFTIPAIISGIKYSKLDYFFIIGISTLSLFLFKMFDSASLI  60
1229492.3_typeI       MELLNSYNFVLFVLTQMILMFTIPAIISGIKYSKLDYFFIIGISTLSLFLFKMFDSASLI  60
1280.4851_typeI       MELLNSYNFVLFVLTQMILMFTIPAIISGIKYSKLDYFFIIGISTLSLFLFKMFDSASLI  60
1006543.3_typeI       MELLNSYNFVLFVLTQMILMFTIPAIISGIKYSKLDYFFIIVISTLSLFLFKMFDSASLI  60
46170.245_typeI       MELLNSYNFVLFVLTQMILMFTIPAIISGIKYSKLDYFFIIGISTLSLFLFKMFDSASLI  60
46170.187_typeI       MELLNSYNFVLFVLTQMILMFTIPAIISGIKYSKLDYFFIIGISTLSLFLFKMFDSASLI  60
1280.5203_typeI       MELLNSYNFVLFVLTQMILMFTIPAIISGIKYSKLDYFFIIGISTLSLFLFKMFDSASLI  60
```

46170.186_typeI	MELLNSYNFVLFVLTQMILMFTIPAIISGIKYSKLDYFFIIGISTLSLFLFKMFDSASLI	60
1193576.3_typeI	MELLNSYNFVLFVLTQMILMFTIPAIISGIKYSKLDYFFIIGISTLSLFLFKMFDSASLI	60
1280.8873_typeI	MELLNSYNFVLFVLTQMILMFTIPAIISVIKYSKLDYFFIIGISTLSLFLFKMFDSASLI	60
46170.185_typeI	MELLNSYNFVLFVLTQMILMFTIPAIISGIKYSKLDYFFIIVISTLSLFLFKMFDSASLI	60
1280.7175_typeI	MELLNSYNFVLFVLTQMILMLTIPAIISGIKYSKLDYFFIIGISTLSLFLFKMFDSASLI	60
1280.7179_typeI	MELLNSYNFVLFVLTQMILMFTIPAIISGIKYSKLDYFFIIGISTLSLFLFKMFDSASLI	60
1280.7178_typeI	MELLNSYNFVLFVLTQMILMFTIPAIISGIKYSKLDYFFIIGISTLSLFLFKMFDSASLI	60
1280.4824_typeI	MELLNSYNFVLFVLTQMILMFTIPAIISGIKYSKLDYFFIIGISTLSLFLFKMFDSASLI	60
1323661.3_typeI	MELLNSYNFVLFVLTQMILMFTIPAIISGIKYSKLDYFFIIGISTLSLFLFKMFDSASLI	60
46170.155_typeI	MELLNSYNFVLFVLTQMILMFTIPAIISGIKYSKLDYFFIIGISTLSLFLFKMFDSASLI	60
1280.3356_typeI	MELLNRYNFVLFVLTQMILMFTIPAIISGIKYSKLDYFFIIVISTLSLFLFKMFDSASLI	60
663951.4_typeI	MELLNRYNFVLFVLTQMILMFTIPAIISGIKYSKLDYFFIIVISTLSLFLFKMFDSASLI	60
1280.7246_typeI	MELLNRYNFVLFVLTQMILMFTIPAIISGIKYSKLDYFFIIVISTLSLFLFKMFDSASLI	60
1280.7245_typeI	MELLNRYNFVLFVLTQMILMFTIPAIISGIKYSKLDYFFIIVISTLSLFLFKMFDSASLI	60
1406863.3_typeI	MELLNRYNFVLFVLTQMILMFTIPAIISGIKYSKLDYFFIIVISTLSLFLFKMFDSASLI	60
1280.3350_typeI	MELLNSYNFVLFVLTQMILMFTIPAIISGIKYSKLDYFFIIVISTLSLFLFKMFDSASLI	60
93062.19_typeI	MELLNSYNFVLFVLTQMILMFTIPAIISGIKYSKLDYFFIIVISTLSLFLFKMFDSASLI	60
1074252.3_typeI	MELLNSYNFVLFVLTQMILMFTIPAIISGIKYSKLDYFFIIVISTLSLFLFKMFDSASLI	60
46170.102_typeI	MELLNSYNFVLFVLTQMILMFTIPAIISGIKYSKLDYFFIIVISTLSLFLFKMFDSASLI	60
1280.3366_typeI	MELLNSYNFVLFVLTQMILMFTIPAIISGIKYSKLDYFFIIVISTLSLFLFKMFDSASLI	60
1280.2220_typeI	MELLNSYNFVLFVLTQMILMFTIPAIISGIKYSKLDYFFIIVISTLSLFLFKMFDSASLI	60
1280.2217_typeI	MELLNSYNFVLFVLTQMILMFTIPAIISGIKYSKLDYFFIIVISTLSLFLFKMFDSASLI	60
1280.2218_typeI	MELLNSYNFVLFVLTQMILMFTIPAIISGIKYSKLDYFFIIVISTLSLFLFKMFDSASLI	60
1280.3574_typeI	MELLNSYNFVLFVLTQMILMFTIPAIISGIKYSKLDYFFIIVISTLSLFLFKMFDSASLI	60
451515.3_typeI	MELLNSYNFVLFVLTQMILMFTIPAIISGIKYSKLDYFFIIVISTLSLFLFKMFDSASLI	60
1458279.3_typeI	MELLNSYNFVLFVLTQMILMFTIPAIISGIKYSKLDYFFIIVISTLSLFLFKMFDSASLI	60
1280.2215_typeI	MELLNSYNFVLFVLTQMILMFTIPAIISGIKYSKLDYFFIIVISTLSLFLFKMFDSASLI	60
46170.148_typeI	MELLNSYNFVLFVLTQMILMFTIPAIISGIKYSKLDYFFIIVISTLSLFLFKMFDSASLI	60
1280.5204_typeI	MELLNSYNFVLFVLTQMILMFTIPAIISGIKYSKLDYFFIIVISTLSLFLFKMFDSASLI	60
1280.4800_typeI	MELLNSYNFVLFVLTQMILMFTIPAIISGIKYSKLDYFFIIVISTLSLFLFKMFDSASLI	60
1280.2216_typeI	MELLNSYNFVLFVLTQMILMFTIPAIISGIKYSKLDYFFIIVISTLSLFLFKMFDSASLI	60
46170.246_typeI	MELLNSYNFVLFVLTQMILMFTIPAIISGIKYSKLDYFFIIVISTLSLFLFKMFDSASLI	60
1280.3352_typeI	MELLNSYNFVLFVLTQMILMFTIPAIISGIKYSKLDYFFIIVISTLSLFLFKMFDSASLI	60
46170.149_typeI	MELLNSYNFVLFVLTQMILMFTIPAIISGIKYSKLDYFFIIVISTLSLFLFKMFDSASLI	60
1280.2219_typeI	MELLNSYNFVLFVLTQMILMFTIPAIISGIKYSKLDYFFIIVISTLSLFLFKMFDSASLI	60
46170.247_typeI	MELLNSYNFVLFVLTQMILMFTIPAIISGIKYSKLDYFFIIVISTLSLFLFKMFDSASLI	60
1280.3566_typeI	MELLNSYNFVLFVLTQMILMFTIPAIISGIKYSKLDYFFIIVISTLSLFLFKMFDSASLI	60
1280.3583_typeI	MELLNSYNFVLFVLTQMILMFTIPAIISGIKYSKLDYFFIIVISTLSLFLFKMFDSASLI	60
451516.9_typeI	MELLNSYNFVLFVLTQMILMFTIPAIISGIKYSKLDYFFIIVISTLSLFLFKMFDSASLI	60
46170.289_typeI	MELLNSYNFVLFVLTQMILMFTIPAIISGIKYSKLDYFFIIVISTLSLFLFKMFDSASLI	60
426430.8_typeI	MELLNSYNFVLFVLTQMILMFTIPAIISGIKYSKLDYFFIIVISTLSLFLFKMFDSASLI	60
1305598.3_typeI	MELLNSYNFVLFVLTQMILMFTIPAIISGIKYSKLDYFFIIVISTLSLFLFKMFDSASLI	60
46170.182_typeI	MELLNSYNFVLFVLTQMILMFTIPAIISGIKYSKLDYFFIIVISTLSLFLFKMFDSASLI	60
46170.290_typeI	MELLNSYNFVLFVLTQMILMFTIPAIISGIKYSKLDYFFIIVISTLSLFLFKMFDSASLI	60
1280.4809_typeI	MELLNSYNFVLFVLTQMILMFTIPAIISGIKYSKLDYFFIIVISTLSLFLFKMFDSASLI	60
1280.3359_typeI	MELLNSYNFVLFVLTQMILMFTIPAIISGIKYSKLDYFFIIVISTLSLFLFKMFDSASLI	60
46170.181_typeI	MELLNSYNFVLFVLTQMILMFTIPAIISGIKYSKLDYFFIIVISTLSLFLFKMFDSASLI	60
46170.183_typeI	MELLNSYNFVLFVLTQMILMFTIPAIISGIKYSKLDYFFIIVISTLSLFLFKMFDSASLI	60
1321369.3_typeI	MELLNSYNFVLFVLTQMILMFTIPAIISGIKYSKLDYFFIIVISTLSLFLFKMFDSASLI	60
1280.10152_typeI	MELLNSYNFVLFVLTQMILMFTIPAIISGIKYSKLDYFFIIVISTLSLFLFKMFDSASLI	60
46170.86_typeI	MELLNSYNFVLFVLTQMILMFTIPAIISGIKYSKLDYFFIIVISTLSLFLFKMFDSASLI	60
1280.4826_typeI	MELLNSYNFVLFVLTQMILMFTIPAIISGIKYSKLDYFFIIVISTLSLFLFKMFDSASLI	60
46170.184_typeI	MELLNSYNFVLFVLTQMILMFTIPAIISGIKYSKLDYFFIIVISTLSLFLFKMFDSASLI	60
1028799.3_typeI	MELLNSYNFVLFVLTQMILMFTIPAIISGIKYSKLDYFFIIVISTLSLFLFKMFDSASLI	60
1280.4849_typeI	MELLNSYNFVLFVLTQMILMFTIPAIISGIKYSKLDYFFIIVISTLSLFLFKMFDSASLI	60
1280.3589_typeI	MELLNSYNFVLFVLTQMILMFTIPAIISGIKYSKLDYFFIIVISTLSLFLFKMFDSASLI	60

```
1241616.6_typeI       MELLNSYNFVLFVLTQMILMFTIPAIISGIKYSKLDYFFIIVISTLSLFLFKMFDSASLI  60
546342.4_typeI        MELLNSYNFVLFVLTQMILMFTIPAIISGIKYSKLDYFFIIVISTLSLFLFKMFDSASLI  60
1280.4805_typeI       MELLNSYNFVLFVLTQMILMFTIPAIISGIKYSKLDYFFIIVISTLSLFLFKMFDSASLI  60
93061.5_typeI         MELLNSYNFVLFVLTQMILMFTIPAIISGIKYSKLDYFFIIVISTLSLFLFKMFDSASLI  60
                      ** ** *******:******:****:** ************ *::*****:*********

1368166.3_typeI       ILTSFIIILYFVKIKWYSILLIMASQIILYCANYMYIVIYAYVTKISDSILVIFPSFLAI  120
1280.10759_typeI      ILTSFIIILYFVKIKWYSILLIMASQIILYCANYMYIVIYAYVTKISDSILVIFPSFLAI  120
1118959.3_typeI       ILTSFIIILYFVKIKWYSILLIMASQIILYCANYMYIVIYAYVTKISDSILVIFPSFLAI  120
685039.3_typeI        ILTSFIIILYFVKIKWYSILLIMASQIILYCANYMYIVIYAYVTKISDSILVIFPSFLAI  120
1201010.3_typeI       ILTSFIIILYFVKIKWYSILLIMASQIILYCANYMYIVIYAYVTKISDSILVIFPSFLAI  120
1194085.3_typeI       ILTSFIIILYFVKIKWYSILLIMASQIILYCANYMYIVIYAYVTKISDSILVIFPSFLAI  120
1280.3367_typeI       ILTSFIIIMYFVKIKWYSILLIMTSQIILYCANYMYIVIFAYITKISDNIFVIFPSFFVV  120
1280.5205_typeI       ILTSFIIIMYFVKIKWYSILLIMTSQIILYCANYMYIVIFAYITKISDNIFVIFPSFFVV  120
523796.5_typeI        ILTSFIIIMYFVKIKWYSILLIMTSQIILYCANYMYIVIYAYITKISDSIFVIFPSFFVV  120
1280.4850_typeI       ILTSFIIIMYFVKIKWYSILLIMTSQIILYCANYMYIVIYAYITKISDSIFVIFPSFFVV  120
1280.4852_typeI       ILTSFIIIMYFVKIKWYSILLIMTSQIILYCANYMYIVIYAYITKISDSIFVIFPSFFVV  120
1155084.3_typeI       ILTSFIIIMYFVKIKWYSILLIMTSQIILYCANYMYIVIYAYITKISDSIFVIFPSFFVV  120
46170.288_typeI       ILTSFIIIMYFVKIKWYSILLIMTSQIILYCANYMYIVIYAYITKISDSIFVIFPSFFVV  120
1229492.3_typeI       ILTSFIIIMYFVKIKWYSILLIMTSQIILYCANYMYIVIYAYITKISDSIFVIFPSFFVV  120
1280.4851_typeI       ILTSFIIIMYFVKIKWYSILLIMTSQIILYCANYMYIVIYAYITKISDSIFVIFPSFFVV  120
1006543.3_typeI       ILTSFIIIMYFVKIKWYSILLIMTSQIILYCANYMYIVIYAYITKISDSIFVIFPSFFVV  120
46170.245_typeI       ILTSFIIIMYFVKIKWYSILLIMTSQIILYCANYMYIVIYAYITKISDSIFVIFPSFFVV  120
46170.187_typeI       ILTSFIIIMYFVKIKWYSILLIMTSQIILYCANYMYIVIYAYITKISDSIFVIFPSFFVV  120
1280.5203_typeI       ILTSFIIIMYFVKIKWYSILLIMTSQIILYCANYMYIVIYAYITKISDSIFVIFPSFFVV  120
46170.186_typeI       ILTSFIIIMYFVKIKWYSILLIMTSQIILYCANYMYIVIYAYITKISDSIFVIFPSFFVV  120
1193576.3_typeI       ILTSFIIIMYFVKIKWYSILLIMTSQIILYCANYMYIVIYAYITKISDSIFVIFPSFFVV  120
1280.8873_typeI       ILTSFIIIMYFVKIKWYSILLIMTSQIILYCANYMYIVIYAYITKISDSIFVIFPSFFVV  120
46170.185_typeI       ILTSFIIIMYFVKIKWYSILLIMTSQIILYCANYMYIVIYAYITKISDSIFVIFPSFFVV  120
1280.7175_typeI       ILTSFIIIMYFVKIKWYSILLIMTSQIILYCANYMYIVIYAYITKISDSIFVIFPSFFVV  120
1280.7179_typeI       ILTSFIIIMYFVKIKWYSILLIMTSQIILYCANYMYIVIYAYITKISDSIFVISPSFFVV  120
1280.7178_typeI       ILTSFIIIMYFVKIKWYSILLIMTSQIILYCANYMYIVIYAYITKISDSIFVISPSFFVV  120
1280.4824_typeI       ILTSFIIIMYFVKIKWYSILLIMTSQIILYCANYMYIVIYAYITKISDSIFVIFPSFFVV  120
1323661.3_typeI       ILTSFIIIMYFVKIKWYSILLIMTSQIILYCANYMYIVIYAYITKISDSIFVIFPSFFVV  120
46170.155_typeI       ILTSFIIIMYFVKIKWYSILLIMTSQIILYCANYMYIVIYAYITKISDSIFVIFPSFFVV  120
1280.3356_typeI       ILTSFIIIMYFVKIKWYSILLIMTSQIILYCANYMYIVIYAYITKISDSIFVIFPSFFVV  120
663951.4_typeI        ILTSFIIIMYFVKIKWYSILLIMTSQIILYCANYMYIVIYAYITKISDSIFVIFPSFFVV  120
1280.7246_typeI       ILTSFIIIMYFVKIKWYSILLIMTSQIILYCANYMYIVIYAYITKISDSIFVIFPSFFVV  120
1280.7245_typeI       ILTSFIIIMYFVKIKWYSILLIMTSQIILYCANYMYIVIYAYITKISDSIFVIFPSFFVV  120
1406863.3_typeI       ILTSFIIIMYFVKIKWYSILLIMTSQIILYCANYMYIVIYAYITKISDSIFVIFPSFFVV  120
1280.3350_typeI       ILTSFIIIMYFVKIKWYSILLIMTSQIILYCANYMYIVIYAYITKISDSIFVIFPSFFVV  120
93062.19_typeI        ILTSFIIIMYFVKIKWYSILLIMTSQIILYCANYMYIVIYAYITKISDSIFVIFPSFFVV  120
1074252.3_typeI       ILTSFIIIMYFVKIKWYSILLIMTSQIILYCANYMYIVIYAYITKISDSIFVIFPSFFVV  120
46170.102_typeI       ILTSFIIIMYFVKIKWYSILLIMTSQIILYCANYMYIVIYAYITKISDSIFVIFPSFFVV  120
1280.3366_typeI       ILTSFIIIMYFVKIKWYSILLIMTSQIILYCANYMYIVIYAYITKISDSIFVIFPSFFVV  120
1280.2220_typeI       ILTSFIIIMYFVKIKWYSILLIMTSQIILYCANYMYIVIYAYITKISDSIFVIFPSFFVV  120
1280.2217_typeI       ILTSFIIIMYFVKIKWYSILLIMTSQIILYCANYMYIVIYAYITKISDSIFVIFPSFFVV  120
1280.2218_typeI       ILTSFIIIMYFVKIKWYSILLIMTSQIILYCANYMYIVIYAYITKISDSIFVIFPSFFVV  120
1280.3574_typeI       ILTSFIIIMYFVKIKWYSILLIMTSQIILYCANYMYIVIYAYITKISDSIFVIFPSFFVV  120
451515.3_typeI        ILTSFIIIMYFVKIKWYSILLIMTSQIILYCANYMYIVIYAYITKISDSIFVIFPSFFVV  120
1458279.3_typeI       ILTSFIIIMYFVKIKWYSILLIMTSQIILYCANYMYIVIYAYITKISDSIFVIFPSFFVV  120
1280.2215_typeI       ILTSFIIIMYFVKIKWYSILLIMTSQIILYCANYMYIVIYAYITKISDSIFVIFPSFFVV  120
46170.148_typeI       ILTSFIIIMYFVKIKWYSILLIMTSQIILYCANYMYIVIYAYITKISDSIFVIFPSFFVV  120
1280.5204_typeI       ILTSFIIIMYFVKIKWYSILLIMTSQIILYCANYMYIVIYAYITKISDSIFVIFPSFFVV  120
1280.4800_typeI       ILTSFIIIMYFVKIKWYSILLIMTSQIILYCANYMYIVIYAYITKISDSIFVIFPSFFVV  120
1280.2216_typeI       ILTSFIIIMYFVKIKWYSILLIMTSQIILYCANYMYIVIYAYITKISDSIFVIFPSFFVV  120
```

```
46170.246_typeI        ILTSFIIIMYFVKIKWYSILLIMTSQIILYCANYMYIVIYAYITKISDSIFVIFPSFFVV  120
1280.3352_typeI        ILTSFIIIMYFVKIKWYSILLIMTSQIILYCANYMYIVIYAYITKISDSIFVIFPSFFVV  120
46170.149_typeI        ILTSFIIIMYFVKIKWYSILLIMTSQIILYCANYMYIVIYAYITKISDSIFVIFPSFFVV  120
1280.2219_typeI        ILTSFIIIMYFVKIKWYSILLIMTSQIILYCANYMYIVIYAYITKISDSIFVIFPSFFVV  120
46170.247_typeI        ILTSFIIIMYFVKIKWYSILLIMTSQIILYCANYMYIVIYAYITKISDSIFVIFPSFFVV  120
1280.3566_typeI        ILTSFIIIMYFVKIKWYSILLIMTSQIILYCANYMYIVIYAYITKISDSIFVIFPSFFVV  120
1280.3583_typeI        ILTSFIIIMYFVKIKWYSILLIMTSQIILYCANYMYIVIYAYITKISDSIFVIFPSFFVV  120
451516.9_typeI         ILTSFIIIMYFVKIKWYSILLIMTSQIILYCANYMYIVIYAYITKISDSIFVIFPSFFVV  120
46170.289_typeI        ILTSFIIIMYFVKIKWYSILLIMTSQIILYCANYMYIVIYAYITKISDSIFVIFPSFFVV  120
426430.8_typeI         ILTSFIIIMYFVKIKWYSILLIMTSQIILYCANYMYIVIYAYITKISDSIFVIFPSFFVV  120
1305598.3_typeI        ILTSFIIIMYFVKIKWYSILLIMTSQIILYCANYMYIVIYAYITKISDSIFVIFPSFFVV  120
46170.182_typeI        ILTSFIIIMYFVKIKWYSILLIMTSQIILYCANYMYIVIYAYITKISDSIFVIFPSFFVV  120
46170.290_typeI        ILTSFIIIMYFVKIKWYSILLIMTSQIILYCANYMYIVIYAYITKISDSIFVIFPSFFVV  120
1280.4809_typeI        ILTSFIIIMYFVKIKWYSILLIMTSQIILYCANYMYIVIYAYITKISDSIFVIFPSFFVV  120
1280.3359_typeI        ILTSFIIIMYFVKIKWYSILLIMTSQIILYCANYMYIVIYAYITKISDSIFVIFPSFFVV  120
46170.181_typeI        ILTSFIIIMYFVKIKWYSILLIMTSQIILYCANYMYIVIYAYITKISDSIFVIFPSFFVV  120
46170.183_typeI        ILTSFIIIMYFVKIKWYSILLIMTSQIILYCANYMYIVIYAYITKISDSIFVIFPSFFVV  120
1321369.3_typeI        ILTSFIIIMYFVKIKWYSILLIMTSQIILYCANYMYIVIYAYITKISDSIFVIFPSFFVV  120
1280.10152_typeI       ILTSFIIIMYFVKIKWYSILLIMTSQIILYCANYMYIVIYAYITKISDSIFVIFPSFFVV  120
46170.86_typeI         ILTSFIIIMYFVKIKWYSILLIMTSQIILYCANYMYIVIYAYITKISDSIFVIFPSFFVV  120
1280.4826_typeI        ILTSFIIIMYFVKIKWYSILLIMTSQIILYCANYMYIVIYAYITKISDSIFVIFPSFFVV  120
46170.184_typeI        ILTSFIIIMYFVKIKWYSILLIMTSQIILYCANYMYIVIYAYITKISDSIFVIFPSFFVV  120
1028799.3_typeI        ILTSFIIIMYFVKIKWYSILLIMTSQIILYCANYMYIVIYAYITKISDSIFVIFPSFFVV  120
1280.4849_typeI        ILTSFIIIMYFVKIKWYSILLIMTSQIILYCANYMYIVIYAYITKISDSIFVIFPSFFVV  120
1280.3589_typeI        ILTSFIIIMYFVKIKWYSILLIMTSQIILYCANYMYIVIYAYITKISDSIFVIFPSFFVV  120
1241616.6_typeI        ILTSFIIIMYFVKIKWYSILLIMTSQIILYCANYMYIVIYAYITKISDSIFVIFPSFFVV  120
546342.4_typeI         ILTSFIIIMYFVKIKWYSILLIMTSQIILYCANYMYIVIYAYITKISDSIFVIFPSFFVV  120
1280.4805_typeI        ILTSFIIIMYFVKIKWYSILLIMTSQIILYCANYMYIVIYAYITKISDSIFVIFPSFFVV  120
93061.5_typeI          ILTSFIIIMYFVKIKWYSILLIMTSQIILYCANYMYIVIYAYITKISDSIFVIFPSFFVV  120
                       ********:*************:**************:**:*****.*:** ***:..:

1368166.3_typeI        YVTISLLFSYIINRVLKKISTSYLILNKGFLIVISTILLLTFSLFFFYSQINSD------  174
1280.10759_typeI       YVTISLLFSYIINRVLKKISTSYLILNKGFLIVISTILLLTFSLFFFYSQINSD------  174
1118959.3_typeI        YVTISLLFSYIINRVLKKISTSYLILNKGFLIVISTILLLTFSLFFFYSQINSD------  174
685039.3_typeI         YVTISLLFSYIINRVLKKISTSYLILNKGFLIVISTILLLTFSLFFFYSQINSD------  174
1201010.3_typeI        YVTISLLFSYIINRVLKKISTSYLILNKGFLIVISTILLLTFSLFFFYSQINSD------  174
1194085.3_typeI        YVTISLLFSYIINRVLKKISTSYLILNKGFLIVISTILLLTFSLFFFYSQINSD------  174
1280.3367_typeI        YVTISLLFSYIINRVLKKISSSYLILNKGFLIVISTILLLTFSLFFFYSQINSD------  174
1280.5205_typeI        YVTISLLFSYIINRVLKKISSSYLILNKGFLIVISTILLLTFSLFFFYSQINSD------  174
523796.5_typeI         YVTISILFSYIINRVLKKISTPYLILNKGFLIVIPTILLLTFSLFFFYSQINSD------  174
1280.4850_typeI        YVTISILFSYIINRVLKKISTPYLILNKGFLIVISTILLLTFSLFFFYSQINSD------  174
1280.4852_typeI        YVTISILFSYIINRVLKKISTPYLILNKGFLIVISTILLLTFSLFFFYSQINSD------  174
1155084.3_typeI        YVTISILFSYIINRVLKKISTPYLILNKGFLIVISTILLLTFSLFFFYSQINSD------  174
46170.288_typeI        YVTISILFSYIINRVLKKISTPYLILNKGFLIVISTILLLTFSLFFFYSQINSD------  174
1229492.3_typeI        YVTISILFSYIINRVLKKISTPYLILNKGFLIVISTILLLTFSLFFFYSQINSD------  174
1280.4851_typeI        YVTISILFSYIINRVLKKISTPYLILNKGFLIVISTILLLTFSLFFFYSQINSD------  174
1006543.3_typeI        YVTISILFSYIINRVLKKISTPYLILNKGFLIVISTILLLTFSLFFFY------------  168
46170.245_typeI        YVTISILFSYIINRVLKKISTPYLILNKGFLIVISTILLLTFSLFFFYSQINSDE-----  175
46170.187_typeI        YVTISILFSYIINRVLKKISTPYLILNKGFLIVISTILLLTFSLFFFYSQINSDE-----  175
1280.5203_typeI        YVTISILFSYIINRVLKKISTPYLILNKGFLIVISTILLLTFSLFFFYSQINSDE-----  175
46170.186_typeI        YVTISILFSYIINRVLKKISTPYLILNKGFLIVISTILLLTFSLFFFYSQINSDE-----  175
1193576.3_typeI        YVTISILFSYIINRVLKKISTPYLILNKGFLIVISTILLLTFSLFFFYSQINSDE-----  175
1280.8873_typeI        YVTISILFSYIINRVLKKISTPYLILNKGFLIVISTILLLTFSLFFFYSQINSDE-----  175
46170.185_typeI        YVTISILFSYIINRVLKKISTPYLILNKGFLIVISTILLLTFSLFFFYSQINSDEAIKSV  180
1280.7175_typeI        YVTISILFSYIINRVLKKISTPYLILNKGFLIVISTILLLTFSLFFFYSQINSDE-----  175
1280.7179_typeI        YVTISILFSYIINRVLKKISTPYLILNKGFLIVISTILLLTFSLFFFYSQINSDE-----  175
```

```
1280.7178_typeI       YVTISILFSYIINRVLKKISTPYLILNKGFLIVISTILLLTFSLFFFYSQINSDE-----  175
1280.4824_typeI       YVTISILFSYIINRVLKKISTPYLILNKGFLIVISTILLLTFSLFFFYSQINSDE-----  175
1323661.3_typeI       YVTISILFSYIINRVLKKISTPYLILNKGFLIVISTILLLTFSLFFFYSQINSDE-----  175
46170.155_typeI       YVTISILFSYIINRVLKKISTPYLILNKGFLIVISTILLLTFSLFFFYSQINSDE-----  175
1280.3356_typeI       YVTISILFSYIINRVLKKISTPYLILNKGFLIVISTILLLTFSLFFFYSQINSDE-----  175
663951.4_typeI        YVTISILFSYIINRVLKKISTPYLILNKGFLIVISTILLLTFSLFFFYSQINSDE-----  175
1280.7246_typeI       YVTISILFSYIINRVLKKISTPYLILNKGFLIVISTILLLTFSLFFFYSQINSDE-----  175
1280.7245_typeI       YVTISILFSYIINRVLKKISTPYLILNKGFLIVISTILLLTFSLFFFYSQINSDE-----  175
1406863.3_typeI       YVTISILFSYIINRVLKKISTPYLILNKGFLIVISTILLLTFSLFFFYSQINSDE-----  175
1280.3350_typeI       YVTISILFSYIINRVLKKISTPYLILNKGFLIVISTILLLTFSLFFFYSQINSDE-----  175
93062.19_typeI        YVTISILFSYIINRVLKKISTPYLILNKGFLIVISTILLLTFSLFFFYSQINSDE-----  175
1074252.3_typeI       YVTISILFSYIINRVLKKISTPYLILNKGFLIVISTILLLTFSLFFFYSQINSDE-----  175
46170.102_typeI       YVTISILFSYIINRVLKKISTPYLILNKGFLIVISTILLLTFSLFFFYSQINSDE-----  175
1280.3366_typeI       YVTISILFSYIINRVLKKISTPYLILNKGFLIVISTILLLTFSLFFFYSQINSDE-----  175
1280.2220_typeI       YVTISILFSYIINRVLKKISTPYLILNKGFLIVISTILLLTFSLFFFYSQINSDE-----  175
1280.2217_typeI       YVTISILFSYIINRVLKKISTPYLILNKGFLIVISTILLLTFSLFFFYSQINSDE-----  175
1280.2218_typeI       YVTISILFSYIINRVLKKISTPYLILNKGFLIVISTILLLTFSLFFFYSQINSDE-----  175
1280.3574_typeI       YVTISILFSYIINRVLKKISTPYLILNKGFLIVISTILLLTFSLFFFYSQINSDE-----  175
451515.3_typeI        YVTISILFSYIINRVLKKISTPYLILNKGFLIVISTILLLTFSLFFFYSQINSDE-----  175
1458279.3_typeI       YVTISILFSYIINRVLKKISTPYLILNKGFLIVISTILLLTFSLFFFYSQINSDE-----  175
1280.2215_typeI       YVTISILFSYIINRVLKKISTPYLILNKGFLIVISTILLLTFSLFFFYSQINSDE-----  175
46170.148_typeI       YVTISILFSYIINRVLKKISTPYLILNKGFLIVISTILLLTFSLFFFYSQINSDE-----  175
1280.5204_typeI       YVTISILFSYIINRVLKKISTPYLILNKGFLIVISTILLLTFSLFFFYSQINSDE-----  175
1280.4800_typeI       YVTISILFSYIINRVLKKISTPYLILNKGFLIVISTILLLTFSLFFFYSQINSDE-----  175
1280.2216_typeI       YVTISILFSYIINRVLKKISTPYLILNKGFLIVISTILLLTFSLFFFYSQINSDE-----  175
46170.246_typeI       YVTISILFSYIINRVLKKISTPYLILNKGFLIVISTILLLTFSLFFFYSQINSDE-----  175
1280.3352_typeI       YVTISILFSYIINRVLKKISTPYLILNKGFLIVISTILLLTFSLFFFYSQINSDE-----  175
46170.149_typeI       YVTISILFSYIINRVLKKISTPYLILNKGFLIVISTILLLTFSLFFFYSQINSDE-----  175
1280.2219_typeI       YVTISILFSYIINRVLKKISTPYLILNKGFLIVISTILLLTFSLFFFYSQINSDE-----  175
46170.247_typeI       YVTISILFSYIINRVLKKISTPYLILNKGFLIVISTILLLTFSLFFFYSQINSDE-----  175
1280.3566_typeI       YVTISILFSYIINRVLKKISTPYLILNKGFLIVISTILLLTFSLFFFYSQINSDE-----  175
1280.3583_typeI       YVTISILFSYIINRVLKKISTPYLILNKGFLIVISTILLLTFSLFFFYSQINSDE-----  175
451516.9_typeI        YVTISILFSYIINRVLKKISTPYLILNKGFLIVISTILLLTFSLFFFYSQINSDE-----  175
46170.289_typeI       YVTISILFSYIINRVLKKISTPYLILNKGFLIVISTILLLTFSLFFFYSQINSDE-----  175
426430.8_typeI        YVTISILFSYIINRVLKKISTPYLILNKGFLIVISTILLLTFSLFFFYSQINSDE-----  175
1305598.3_typeI       YVTISILFSYIINRVLKKISTPYLILNKGFLIVISTILLLTFSLFFFYSQINSDE-----  175
46170.182_typeI       YVTISILFSYIINRVLKKISTPYLILNKGFLIVISTILLLTFSLFFFYSQINSDE-----  175
46170.290_typeI       YVTISILFSYIINRVLKKISTPYLILNKGFLIVISTILLLTFSLFFFYSQINSDE-----  175
1280.4809_typeI       YVTISILFSYIINRVLKKISTPYLILNKGFLIVISTILLLTFSLFFFYSQINSDE-----  175
1280.3359_typeI       YVTISILFSYIINRVLKKISTPYLILNKGFLIVISTILLLTFSLFFFYSQINSDE-----  175
46170.181_typeI       YVTISILFSYIINRVLKKISTPYLILNKGFLIVISTILLLTFSLFFFYSQINSDE-----  175
46170.183_typeI       YVTISILFSYIINRVLKKISTPYLILNKGFLIVISTILLLTFSLFFFYSQINSDE-----  175
1321369.3_typeI       YVTISILFSYIINRVLKKISTPYLILNKGFLIVISTILLLTFSLFFFYSQINSDE-----  175
1280.10152_typeI      YVTISILFSYIINRVLKKISTPYLILNKGFLIVISTILLLTFSLFFFYSQINSDE-----  175
46170.86_typeI        YVTISILFSYIINRVLKKISTPYLILNKGFLIVISTILLLTFSLFFFYSQINSDE-----  175
1280.4826_typeI       YVTISILFSYIINRVLKKISTPYLILNKGFLIVISTILLLTFSLFFFYSQINSDE-----  175
46170.184_typeI       YVTISILFSYIINRVLKKISTPYLILNKGFLIVISTILLLTFSLFFFYSQINSDE-----  175
1028799.3_typeI       YVTISILFSYIINRVLKKISTPYLILNKGFLIVISTILLLTFSLFFFYSQINSDE-----  175
1280.4849_typeI       YVTISILFSYIINRVLKKISTPYLILNKGFLIVISTILLLTFSLFFFYSQINSDE-----  175
1280.3589_typeI       YVTISILFSYIINRVLKKISTPYLILNKGFLIVISTILLLTFSLFFFYSQINSDE-----  175
1241616.6_typeI       YVTISILFSYIINRVLKKISTPYLILNKGFLIVISTILLLTFSLFFFYSQINSDE-----  175
546342.4_typeI        YVTISILFSYIINRVLKKISTPYLILNKGFLIVISTILLLTFSLFFFYSQINSDE-----  175
1280.4805_typeI       YVTISILFSYIINRVLKKISTPYLILNKGFLIVISTILLLTFSLFFFYSQINSDE-----  175
93061.5_typeI         YVTISILFSYIINRVLKKISTPYLILNKGFLIVISTILLLTFSLFFFYSQINSDE-----  175
                      *****:**************: ************ *************
```

```
1368166.3_typeI       ------------------------------------------------------------  174
1280.10759_typeI      ------------------------------------------------------------  174
1118959.3_typeI       ------------------------------------------------------------  174
685039.3_typeI        ------------------------------------------------------------  174
1201010.3_typeI       ------------------------------------------------------------  174
1194085.3_typeI       ------------------------------------------------------------  174
1280.3367_typeI       ------------------------------------------------------------  174
1280.5205_typeI       ------------------------------------------------------------  174
523796.5_typeI        ------------------------------------------------------------  174
1280.4850_typeI       ------------------------------------------------------------  174
1280.4852_typeI       ------------------------------------------------------------  174
1155084.3_typeI       ------------------------------------------------------------  174
46170.288_typeI       ------------------------------------------------------------  174
1229492.3_typeI       ------------------------------------------------------------  174
1280.4851_typeI       ------------------------------------------------------------  174
1006543.3_typeI       MTQVHFTLKSEEIQSIIEYSVKDDVSKNILTTVFNQLMENQRTEYIQAKEYERTENRQSQ  228
46170.245_typeI       ------------------------------------------------------------  175
46170.187_typeI       ------------------------------------------------------------  175
1280.5203_typeI       ------------------------------------------------------------  175
46170.186_typeI       ------------------------------------------------------------  175
1193576.3_typeI       ------------------------------------------------------------  175
1280.8873_typeI       ------------------------------------------------------------  175
46170.185_typeI       MTQVHFTLKSEEIQSIIEYSVKDDVSKNILTTVFNQLMENQRTEYIQAKEYERTENRQSQ  240
1280.7175_typeI       ------------------------------------------------------------  175
1280.7179_typeI       ------------------------------------------------------------  175
1280.7178_typeI       ------------------------------------------------------------  175
1280.4824_typeI       ------------------------------------------------------------  175
1323661.3_typeI       ------------------------------------------------------------  175
46170.155_typeI       ------------------------------------------------------------  175
1280.3356_typeI       ------------------------------------------------------------  175
663951.4_typeI        ------------------------------------------------------------  175
1280.7246_typeI       ------------------------------------------------------------  175
1280.7245_typeI       ------------------------------------------------------------  175
1406863.3_typeI       ------------------------------------------------------------  175
1280.3350_typeI       ------------------------------------------------------------  175
93062.19_typeI        ------------------------------------------------------------  175
1074252.3_typeI       ------------------------------------------------------------  175
46170.102_typeI       ------------------------------------------------------------  175
1280.3366_typeI       ------------------------------------------------------------  175
1280.2220_typeI       ------------------------------------------------------------  175
1280.2217_typeI       ------------------------------------------------------------  175
1280.2218_typeI       ------------------------------------------------------------  175
1280.3574_typeI       ------------------------------------------------------------  175
451515.3_typeI        ------------------------------------------------------------  175
1458279.3_typeI       ------------------------------------------------------------  175
1280.2215_typeI       ------------------------------------------------------------  175
46170.148_typeI       ------------------------------------------------------------  175
1280.5204_typeI       ------------------------------------------------------------  175
1280.4800_typeI       ------------------------------------------------------------  175
1280.2216_typeI       ------------------------------------------------------------  175
46170.246_typeI       ------------------------------------------------------------  175
1280.3352_typeI       ------------------------------------------------------------  175
46170.149_typeI       ------------------------------------------------------------  175
1280.2219_typeI       ------------------------------------------------------------  175
46170.247_typeI       ------------------------------------------------------------  175
1280.3566_typeI       ------------------------------------------------------------  175
```

```
1280.3583_typeI       ------------------------------------------------------------ 175
451516.9_typeI        ------------------------------------------------------------ 175
46170.289_typeI       ------------------------------------------------------------ 175
426430.8_typeI        ------------------------------------------------------------ 175
1305598.3_typeI       ------------------------------------------------------------ 175
46170.182_typeI       ------------------------------------------------------------ 175
46170.290_typeI       ------------------------------------------------------------ 175
1280.4809_typeI       ------------------------------------------------------------ 175
1280.3359_typeI       ------------------------------------------------------------ 175
46170.181_typeI       ------------------------------------------------------------ 175
46170.183_typeI       ------------------------------------------------------------ 175
1321369.3_typeI       ------------------------------------------------------------ 175
1280.10152_typeI      ------------------------------------------------------------ 175
46170.86_typeI        ------------------------------------------------------------ 175
1280.4826_typeI       ------------------------------------------------------------ 175
46170.184_typeI       ------------------------------------------------------------ 175
1028799.3_typeI       ------------------------------------------------------------ 175
1280.4849_typeI       ------------------------------------------------------------ 175
1280.3589_typeI       ------------------------------------------------------------ 175
1241616.6_typeI       ------------------------------------------------------------ 175
546342.4_typeI        ------------------------------------------------------------ 175
1280.4805_typeI       ------------------------------------------------------------ 175
93061.5_typeI         ------------------------------------------------------------ 175


1368166.3_typeI       ------------------------------------------------------------ 174
1280.10759_typeI      ------------------------------------------------------------ 174
1118959.3_typeI       ------------------------------------------------------------ 174
685039.3_typeI        ------------------------------------------------------------ 174
1201010.3_typeI       ------------------------------------------------------------ 174
1194085.3_typeI       ------------------------------------------------------------ 174
1280.3367_typeI       ------------------------------------------------------------ 174
1280.5205_typeI       ------------------------------------------------------------ 174
523796.5_typeI        ------------------------------------------------------------ 174
1280.4850_typeI       ------------------------------------------------------------ 174
1280.4852_typeI       ------------------------------------------------------------ 174
1155084.3_typeI       ------------------------------------------------------------ 174
46170.288_typeI       ------------------------------------------------------------ 174
1229492.3_typeI       ------------------------------------------------------------ 174
1280.4851_typeI       ------------------------------------------------------------ 174
1006543. 3_typeI       RNGYYERSFTTRVGTLELKVPRTRDGHFSPTVFERYQRNEKALMASMLEMYVSGVSTRKV 288
46170.245_typeI       ------------------------------------------------------------ 175
46170.187_typeI       ------------------------------------------------------------ 175
1280.5203_typeI       ------------------------------------------------------------ 175
46170.186_typeI       ------------------------------------------------------------ 175
1193576.3_typeI       ------------------------------------------------------------ 175
1280.8873_typeI       ------------------------------------------------------------ 175
46170.185_typeI       RNGYYERSFTTRVGTLELKVPRTRDGHFSPTVFERYQRNEKALMASMLEMYVSGVSTRKV 300
1280.7175_typeI       ------------------------------------------------------------ 175
1280.7179_typeI       ------------------------------------------------------------ 175
1280.7178_typeI       ------------------------------------------------------------ 175
1280.4824_typeI       ------------------------------------------------------------ 175
1323661.3_typeI       ------------------------------------------------------------ 175
46170.155_typeI       ------------------------------------------------------------ 175
1280.3356_typeI       ------------------------------------------------------------ 175
663951.4_typeI        ------------------------------------------------------------ 175
```

```
1280.7246_typeI        ------------------------------------------------------------  175
1280.7245_typeI        ------------------------------------------------------------  175
1406863.3_typeI        ------------------------------------------------------------  175
1280.3350_typeI        ------------------------------------------------------------  175
93062.19_typeI         ------------------------------------------------------------  175
1074252.3_typeI        ------------------------------------------------------------  175
46170.102_typeI        ------------------------------------------------------------  175
1280.3366_typeI        ------------------------------------------------------------  175
1280.2220_typeI        ------------------------------------------------------------  175
1280.2217_typeI        ------------------------------------------------------------  175
1280.2218_typeI        ------------------------------------------------------------  175
1280.3574_typeI        ------------------------------------------------------------  175
451515.3_typeI         ------------------------------------------------------------  175
1458279.3_typeI        ------------------------------------------------------------  175
1280.2215_typeI        ------------------------------------------------------------  175
46170.148_typeI        ------------------------------------------------------------  175
1280.5204_typeI        ------------------------------------------------------------  175
1280.4800_typeI        ------------------------------------------------------------  175
1280.2216_typeI        ------------------------------------------------------------  175
46170.246_typeI        ------------------------------------------------------------  175
1280.3352_typeI        ------------------------------------------------------------  175
46170.149_typeI        ------------------------------------------------------------  175
1280.2219_typeI        ------------------------------------------------------------  175
46170.247_typeI        ------------------------------------------------------------  175
1280.3566_typeI        ------------------------------------------------------------  175
1280.3583_typeI        ------------------------------------------------------------  175
451516.9_typeI         ------------------------------------------------------------  175
46170.289_typeI        ------------------------------------------------------------  175
426430.8_typeI         ------------------------------------------------------------  175
1305598.3_typeI        ------------------------------------------------------------  175
46170.182_typeI        ------------------------------------------------------------  175
46170.290_typeI        ------------------------------------------------------------  175
1280.4809_typeI        ------------------------------------------------------------  175
1280.3359_typeI        ------------------------------------------------------------  175
46170.181_typeI        ------------------------------------------------------------  175
46170.183_typeI        ------------------------------------------------------------  175
1321369.3_typeI        ------------------------------------------------------------  175
1280.10152_typeI       ------------------------------------------------------------  175
46170.86_typeI         ------------------------------------------------------------  175
1280.4826_typeI        ------------------------------------------------------------  175
46170.184_typeI        ------------------------------------------------------------  175
1028799.3_typeI        ------------------------------------------------------------  175
1280.4849_typeI        ------------------------------------------------------------  175
1280.3589_typeI        ------------------------------------------------------------  175
1241616.6_typeI        ------------------------------------------------------------  175
546342.4_typeI         ------------------------------------------------------------  175
1280.4805_typeI        ------------------------------------------------------------  175
93061.5_typeI          ------------------------------------------------------------  175


1368166.3_typeI        ------------------------------------------------------------  174
1280.10759_typeI       ------------------------------------------------------------  174
1118959.3_typeI        ------------------------------------------------------------  174
685039.3_typeI         ------------------------------------------------------------  174
1201010.3_typeI        ------------------------------------------------------------  174
1194085.3_typeI        ------------------------------------------------------------  174
```

```
1280.3367_typeI      ------------------------------------------------------------  174
1280.5205_typeI      ------------------------------------------------------------  174
523796.5_typeI       ------------------------------------------------------------  174
1280.4850_typeI      ------------------------------------------------------------  174
1280.4852_typeI      ------------------------------------------------------------  174
1155084.3_typeI      ------------------------------------------------------------  174
46170.288_typeI      ------------------------------------------------------------  174
1229492.3_typeI      ------------------------------------------------------------  174
1280.4851_typeI      ------------------------------------------------------------  174
1006543.3_typeI      SKIVEELCGKSVSKSFVSSLTEQLEPMVSEWQNRLLSEKNYPYLMTDVLYIKVREENRVL  348
46170.245_typeI      ------------------------------------------------------------  175
46170.187_typeI      ------------------------------------------------------------  175
1280.5203_typeI      ------------------------------------------------------------  175
46170.186_typeI      ------------------------------------------------------------  175
1193576.3_typeI      ------------------------------------------------------------  175
1280.8873_typeI      ------------------------------------------------------------  175
46170.185_typeI      SKIVEELCGKSVSKSFVSSLTEQLEPMVNEWQNRLLSEKNYPYLMTDVLYIKVREENRVL  360
1280.7175_typeI      ------------------------------------------------------------  175
1280.7179_typeI      ------------------------------------------------------------  175
1280.7178_typeI      ------------------------------------------------------------  175
1280.4824_typeI      ------------------------------------------------------------  175
1323661.3_typeI      ------------------------------------------------------------  175
46170.155_typeI      ------------------------------------------------------------  175
1280.3356_typeI      ------------------------------------------------------------  175
663951.4_typeI       ------------------------------------------------------------  175
1280.7246_typeI      ------------------------------------------------------------  175
1280.7245_typeI      ------------------------------------------------------------  175
1406863.3_typeI      ------------------------------------------------------------  175
1280.3350_typeI      ------------------------------------------------------------  175
93062.19_typeI       ------------------------------------------------------------  175
1074252.3_typeI      ------------------------------------------------------------  175
46170.102_typeI      ------------------------------------------------------------  175
1280.3366_typeI      ------------------------------------------------------------  175
1280.2220_typeI      ------------------------------------------------------------  175
1280.2217_typeI      ------------------------------------------------------------  175
1280.2218_typeI      ------------------------------------------------------------  175
1280.3574_typeI      ------------------------------------------------------------  175
451515.3_typeI       ------------------------------------------------------------  175
1458279.3_typeI      ------------------------------------------------------------  175
1280.2215_typeI      ------------------------------------------------------------  175
46170.148_typeI      ------------------------------------------------------------  175
1280.5204_typeI      ------------------------------------------------------------  175
1280.4800_typeI      ------------------------------------------------------------  175
1280.2216_typeI      ------------------------------------------------------------  175
46170.246_typeI      ------------------------------------------------------------  175
1280.3352_typeI      ------------------------------------------------------------  175
46170.149_typeI      ------------------------------------------------------------  175
1280.2219_typeI      ------------------------------------------------------------  175
46170.247_typeI      ------------------------------------------------------------  175
1280.3566_typeI      ------------------------------------------------------------  175
1280.3583_typeI      ------------------------------------------------------------  175
451516.9_typeI       ------------------------------------------------------------  175
46170.289_typeI      ------------------------------------------------------------  175
426430.8_typeI       ------------------------------------------------------------  175
1305598.3_typeI      ------------------------------------------------------------  175
46170.182_typeI      ------------------------------------------------------------  175
```

```
46170.290_typeI      ------------------------------------------------------------ 175
1280.4809_typeI      ------------------------------------------------------------ 175
1280.3359_typeI      ------------------------------------------------------------ 175
46170.181_typeI      ------------------------------------------------------------ 175
46170.183_typeI      ------------------------------------------------------------ 175
1321369.3_typeI      ------------------------------------------------------------ 175
1280.10152_typeI     ------------------------------------------------------------ 175
46170.86_typeI       ------------------------------------------------------------ 175
1280.4826_typeI      ------------------------------------------------------------ 175
46170.184_typeI      ------------------------------------------------------------ 175
1028799.3_typeI      ------------------------------------------------------------ 175
1280.4849_typeI      ------------------------------------------------------------ 175
1280.3589_typeI      ------------------------------------------------------------ 175
1241616.6_typeI      ------------------------------------------------------------ 175
546342.4_typeI       ------------------------------------------------------------ 175
1280.4805_typeI      ------------------------------------------------------------ 175
93061.5_typeI        ------------------------------------------------------------ 175


1368166.3_typeI      ------------------------------------------------------------ 174
1280.10759_typeI     ------------------------------------------------------------ 174
1118959.3_typeI      ------------------------------------------------------------ 174
685039.3_typeI       ------------------------------------------------------------ 174
1201010.3_typeI      ------------------------------------------------------------ 174
1194085.3_typeI      ------------------------------------------------------------ 174
1280.3367_typeI      ------------------------------------------------------------ 174
1280.5205_typeI      ------------------------------------------------------------ 174
523796.5_typeI       ------------------------------------------------------------ 174
1280.4850_typeI      ------------------------------------------------------------ 174
1280.4852_typeI      ------------------------------------------------------------ 174
1155084.3_typeI      ------------------------------------------------------------ 174
46170.288_typeI      ------------------------------------------------------------ 174
1229492.3_typeI      ------------------------------------------------------------ 174
1280.4851_typeI      ------------------------------------------------------------ 174
1006543.3_typeI      SKSCHIAIGITKDGDREIIGFMIQSGESEETWTTFFEYLKERGLQGTELVISDAHKGLVS 408
46170.245_typeI      ------------------------------------------------------------ 175
46170.187_typeI      ------------------------------------------------------------ 175
1280.5203_typeI      ------------------------------------------------------------ 175
46170.186_typeI      ------------------------------------------------------------ 175
1193576.3_typeI      ------------------------------------------------------------ 175
1280.8873_typeI      ------------------------------------------------------------ 175
46170.185_typeI      SKSCHIAIGITKDGDREIIGFMIQSGESEETWTTFFEYLKERGLQGTELVISDAHKGLVS 420
1280.7175_typeI      ------------------------------------------------------------ 175
1280.7179_typeI      ------------------------------------------------------------ 175
1280.7178_typeI      ------------------------------------------------------------ 175
1280.4824_typeI      ------------------------------------------------------------ 175
1323661.3_typeI      ------------------------------------------------------------ 175
46170.155_typeI      ------------------------------------------------------------ 175
1280.3356_typeI      ------------------------------------------------------------ 175
663951.4_typeI       ------------------------------------------------------------ 175
1280.7246_typeI      ------------------------------------------------------------ 175
1280.7245_typeI      ------------------------------------------------------------ 175
1406863.3_typeI      ------------------------------------------------------------ 175
1280.3350_typeI      ------------------------------------------------------------ 175
93062.19_typeI       ------------------------------------------------------------ 175
1074252.3_typeI      ------------------------------------------------------------ 175
```

```
46170.102_typeI       ------------------------------------------------------------  175
1280.3366_typeI       ------------------------------------------------------------  175
1280.2220_typeI       ------------------------------------------------------------  175
1280.2217_typeI       ------------------------------------------------------------  175
1280.2218_typeI       ------------------------------------------------------------  175
1280.3574_typeI       ------------------------------------------------------------  175
451515.3_typeI        ------------------------------------------------------------  175
1458279.3_typeI       ------------------------------------------------------------  175
1280.2215_typeI       ------------------------------------------------------------  175
46170.148_typeI       ------------------------------------------------------------  175
1280.5204_typeI       ------------------------------------------------------------  175
1280.4800_typeI       ------------------------------------------------------------  175
1280.2216_typeI       ------------------------------------------------------------  175
46170.246_typeI       ------------------------------------------------------------  175
1280.3352_typeI       ------------------------------------------------------------  175
46170.149_typeI       ------------------------------------------------------------  175
1280.2219_typeI       ------------------------------------------------------------  175
46170.247_typeI       ------------------------------------------------------------  175
1280.3566_typeI       ------------------------------------------------------------  175
1280.3583_typeI       ------------------------------------------------------------  175
451516.9_typeI        ------------------------------------------------------------  175
46170.289_typeI       ------------------------------------------------------------  175
426430.8_typeI        ------------------------------------------------------------  175
1305598.3_typeI       ------------------------------------------------------------  175
46170.182_typeI       ------------------------------------------------------------  175
46170.290_typeI       ------------------------------------------------------------  175
1280.4809_typeI       ------------------------------------------------------------  175
1280.3359_typeI       ------------------------------------------------------------  175
46170.181_typeI       ------------------------------------------------------------  175
46170.183_typeI       ------------------------------------------------------------  175
1321369.3_typeI       ------------------------------------------------------------  175
1280.10152_typeI      ------------------------------------------------------------  175
46170.86_typeI        ------------------------------------------------------------  175
1280.4826_typeI       ------------------------------------------------------------  175
46170.184_typeI       ------------------------------------------------------------  175
1028799.3_typeI       ------------------------------------------------------------  175
1280.4849_typeI       ------------------------------------------------------------  175
1280.3589_typeI       ------------------------------------------------------------  175
1241616.6_typeI       ------------------------------------------------------------  175
546342.4_typeI        ------------------------------------------------------------  175
1280.4805_typeI       ------------------------------------------------------------  175
93061.5_typeI         ------------------------------------------------------------  175


1368166.3_typeI       ------------------------------------------------------------  174
1280.10759_typeI      ------------------------------------------------------------  174
1118959.3_typeI       ------------------------------------------------------------  174
685039.3_typeI        ------------------------------------------------------------  174
1201010.3_typeI       ------------------------------------------------------------  174
1194085.3_typeI       ------------------------------------------------------------  174
1280.3367_typeI       ------------------------------------------------------------  174
1280.5205_typeI       ------------------------------------------------------------  174
523796.5_typeI        ------------------------------------------------------------  174
1280.4850_typeI       ------------------------------------------------------------  174
1280.4852_typeI       ------------------------------------------------------------  174
1155084.3_typeI       ------------------------------------------------------------  174
```

```
46170.288_typeI      ------------------------------------------------------------ 174
1229492.3_typeI      ------------------------------------------------------------ 174
1280.4851_typeI      ------------------------------------------------------------ 174
1006543.3_typeI      AIRKSFTNVSWQRCQVHFLRNIFTTIPKKNSKSFREAVKGIFKFTDINLAREAKNRLIHD 468
46170.245_typeI      ------------------------------------------------------------ 175
46170.187_typeI      ------------------------------------------------------------ 175
1280.5203_typeI      ------------------------------------------------------------ 175
46170.186_typeI      ------------------------------------------------------------ 175
1193576.3_typeI      ------------------------------------------------------------ 175
1280.8873_typeI      ------------------------------------------------------------ 175
46170.185_typeI      AIRKSFTNVSWQRCQVHFLRNIFTTIPKKNSKSFREAVKGIFKFTDINLAREAKNRLIHD 480
1280.7175_typeI      ------------------------------------------------------------ 175
1280.7179_typeI      ------------------------------------------------------------ 175
1280.7178_typeI      ------------------------------------------------------------ 175
1280.4824_typeI      ------------------------------------------------------------ 175
1323661.3_typeI      ------------------------------------------------------------ 175
46170.155_typeI      ------------------------------------------------------------ 175
1280.3356_typeI      ------------------------------------------------------------ 175
663951.4_typeI       ------------------------------------------------------------ 175
1280.7246_typeI      ------------------------------------------------------------ 175
1280.7245_typeI      ------------------------------------------------------------ 175
1406863.3_typeI      ------------------------------------------------------------ 175
1280.3350_typeI      ------------------------------------------------------------ 175
93062.19_typeI       ------------------------------------------------------------ 175
1074252.3_typeI      ------------------------------------------------------------ 175
46170.102_typeI      ------------------------------------------------------------ 175
1280.3366_typeI      ------------------------------------------------------------ 175
1280.2220_typeI      ------------------------------------------------------------ 175
1280.2217_typeI      ------------------------------------------------------------ 175
1280.2218_typeI      ------------------------------------------------------------ 175
1280.3574_typeI      ------------------------------------------------------------ 175
451515.3_typeI       ------------------------------------------------------------ 175
1458279.3_typeI      ------------------------------------------------------------ 175
1280.2215_typeI      ------------------------------------------------------------ 175
46170.148_typeI      ------------------------------------------------------------ 175
1280.5204_typeI      ------------------------------------------------------------ 175
1280.4800_typeI      ------------------------------------------------------------ 175
1280.2216_typeI      ------------------------------------------------------------ 175
46170.246_typeI      ------------------------------------------------------------ 175
1280.3352_typeI      ------------------------------------------------------------ 175
46170.149_typeI      ------------------------------------------------------------ 175
1280.2219_typeI      ------------------------------------------------------------ 175
46170.247_typeI      ------------------------------------------------------------ 175
1280.3566_typeI      ------------------------------------------------------------ 175
1280.3583_typeI      ------------------------------------------------------------ 175
451516.9_typeI       ------------------------------------------------------------ 175
46170.289_typeI      ------------------------------------------------------------ 175
426430.8_typeI       ------------------------------------------------------------ 175
1305598.3_typeI      ------------------------------------------------------------ 175
46170.182_typeI      ------------------------------------------------------------ 175
46170.290_typeI      ------------------------------------------------------------ 175
1280.4809_typeI      ------------------------------------------------------------ 175
1280.3359_typeI      ------------------------------------------------------------ 175
46170.181_typeI      ------------------------------------------------------------ 175
46170.183_typeI      ------------------------------------------------------------ 175
1321369.3_typeI      ------------------------------------------------------------ 175
```

```
1280.10152_typeI     ------------------------------------------------------------ 175
46170.86_typeI       ------------------------------------------------------------ 175
1280.4826_typeI      ------------------------------------------------------------ 175
46170.184_typeI      ------------------------------------------------------------ 175
1028799.3_typeI      ------------------------------------------------------------ 175
1280.4849_typeI      ------------------------------------------------------------ 175
1280.3589_typeI      ------------------------------------------------------------ 175
1241616.6_typeI      ------------------------------------------------------------ 175
546342.4_typeI       ------------------------------------------------------------ 175
1280.4805_typeI      ------------------------------------------------------------ 175
93061.5_typeI        ------------------------------------------------------------ 175


1368166.3_typeI      ------------------------------------------------------------ 174
1280.10759_typeI     ------------------------------------------------------------ 174
1118959.3_typeI      ------------------------------------------------------------ 174
685039.3_typeI       ------------------------------------------------------------ 174
1201010.3_typeI      ------------------------------------------------------------ 174
1194085.3_typeI      ------------------------------------------------------------ 174
1280.3367_typeI      ------------------------------------------------------------ 174
1280.5205_typeI      ------------------------------------------------------------ 174
523796.5_typeI       ------------------------------------------------------------ 174
1280.4850_typeI      ------------------------------------------------------------ 174
1280.4852_typeI      ------------------------------------------------------------ 174
1155084.3_typeI      ------------------------------------------------------------ 174
46170.288_typeI      ------------------------------------------------------------ 174
1229492.3_typeI      ------------------------------------------------------------ 174
1280.4851_typeI      ------------------------------------------------------------ 174
1006543.3_typeI      YIDQPKYSKACASLDDGFEDAFQYTVQGNSHNRLKSTNLIERLNQEVRRREKIIRIFPNQ 528
46170.245_typeI      ------------------------------------------------------------ 175
46170.187_typeI      ------------------------------------------------------------ 175
1280.5203_typeI      ------------------------------------------------------------ 175
46170.186_typeI      ------------------------------------------------------------ 175
1193576.3_typeI      ------------------------------------------------------------ 175
1280.8873_typeI      ------------------------------------------------------------ 175
46170.185_typeI      YIDQPKYSKACASLDDGFEDAFQYTVQGNSHNRLKSTNLIERLNQEVRRREKIIRIFPNQ 540
1280.7175_typeI      ------------------------------------------------------------ 175
1280.7179_typeI      ------------------------------------------------------------ 175
1280.7178_typeI      ------------------------------------------------------------ 175
1280.4824_typeI      ------------------------------------------------------------ 175
1323661.3_typeI      ------------------------------------------------------------ 175
46170.155_typeI      ------------------------------------------------------------ 175
1280.3356_typeI      ------------------------------------------------------------ 175
663951.4_typeI       ------------------------------------------------------------ 175
1280.7246_typeI      ------------------------------------------------------------ 175
1280.7245_typeI      ------------------------------------------------------------ 175
1406863.3_typeI      ------------------------------------------------------------ 175
1280.3350_typeI      ------------------------------------------------------------ 175
93062.19_typeI       ------------------------------------------------------------ 175
1074252.3_typeI      ------------------------------------------------------------ 175
46170.102_typeI      ------------------------------------------------------------ 175
1280.3366_typeI      ------------------------------------------------------------ 175
1280.2220_typeI      ------------------------------------------------------------ 175
1280.2217_typeI      ------------------------------------------------------------ 175
1280.2218_typeI      ------------------------------------------------------------ 175
1280.3574_typeI      ------------------------------------------------------------ 175
```

```
451515.3_typeI        ------------------------------------------------------------  175
1458279.3_typeI       ------------------------------------------------------------  175
1280.2215_typeI       ------------------------------------------------------------  175
46170.148_typeI       ------------------------------------------------------------  175
1280.5204_typeI       ------------------------------------------------------------  175
1280.4800_typeI       ------------------------------------------------------------  175
1280.2216_typeI       ------------------------------------------------------------  175
46170.246_typeI       ------------------------------------------------------------  175
1280.3352_typeI       ------------------------------------------------------------  175
46170.149_typeI       ------------------------------------------------------------  175
1280.2219_typeI       ------------------------------------------------------------  175
46170.247_typeI       ------------------------------------------------------------  175
1280.3566_typeI       ------------------------------------------------------------  175
1280.3583_typeI       ------------------------------------------------------------  175
451516.9_typeI        ------------------------------------------------------------  175
46170.289_typeI       ------------------------------------------------------------  175
426430.8_typeI        ------------------------------------------------------------  175
1305598.3_typeI       ------------------------------------------------------------  175
46170.182_typeI       ------------------------------------------------------------  175
46170.290_typeI       ------------------------------------------------------------  175
1280.4809_typeI       ------------------------------------------------------------  175
1280.3359_typeI       ------------------------------------------------------------  175
46170.181_typeI       ------------------------------------------------------------  175
46170.183_typeI       ------------------------------------------------------------  175
1321369.3_typeI       ------------------------------------------------------------  175
1280.10152_typeI      ------------------------------------------------------------  175
46170.86_typeI        ------------------------------------------------------------  175
1280.4826_typeI       ------------------------------------------------------------  175
46170.184_typeI       ------------------------------------------------------------  175
1028799.3_typeI       ------------------------------------------------------------  175
1280.4849_typeI       ------------------------------------------------------------  175
1280.3589_typeI       ------------------------------------------------------------  175
1241616.6_typeI       ------------------------------------------------------------  175
546342.4_typeI        ------------------------------------------------------------  175
1280.4805_typeI       ------------------------------------------------------------  175
93061.5_typeI         ------------------------------------------------------------  175


1368166.3_typeI       ---------------------------------EAKVIRQYSFIFIGITIFLSILTFVIS  201
1280.10759_typeI      ---------------------------------EAKVIRQYSFIFIGITIFLSILTFVIS  201
1118959.3_typeI       ---------------------------------EAKVIRQYSFIFIGITIFLSILTFVIS  201
685039.3_typeI        ---------------------------------EAKVIRQYSFIFIGITIFLSILTFVIS  201
1201010.3_typeI       ---------------------------------EAKVIRQYSFIFIGITIFLSILTFVIS  201
1194085.3_typeI       ---------------------------------EAKVIRQYSFIFIGITIFLSILTFVIS  201
1280.3367_typeI       ---------------------------------EAKVIRQYSFIFIGITIFLSILTFVIS  201
1280.5205_typeI       ---------------------------------EAKVIRQYSFIFIGITIFLSILTFVIS  201
523796.5_typeI        ---------------------------------EAKVIRQYSFIFIGITIFLSILTFVIS  201
1280.4850_typeI       ---------------------------------EAKVIRQYSFIFIGITIFLSILTFVIS  201
1280.4852_typeI       ---------------------------------EAKVIRQYSFIFIGITIFLSILTFVIS  201
1155084.3_typeI       ---------------------------------EAKVIRQYSFIFIGITIFLSILTFVIS  201
46170.288_typeI       ---------------------------------EAKVIRQYSFIFIGITIFLSILTFVIS  201
1229492.3_typeI       ---------------------------------EAKVIRQYSFIFIGITIFLSILTFVIS  201
1280.4851_typeI       ---------------------------------EARVIRQYSFIFIGITIFLSILTFVIS  201
1006543.3_typeI       TSANRLIGAVLMDLHDEWIYSSRKYINFDK------------------------------  558
46170.245_typeI       ----------------------------------TKVIRQYSFIFIGITIFLSILTFVIS  201
46170.187_typeI       ----------------------------------AKVIRQYSFIFIGITIFLSILTFVIS  201
```

```
1280.5203_typeI        ----------------------------------AKVIRQYSFIFIGITIFLSILTFVIS  201
46170.186_typeI        ----------------------------------AKVIRQYSFIFIGITIFLSILTFVIS  201
1193576.3_typeI        ----------------------------------AKVIRQYSFIFIGITIFLSILTFVIS  201
1280.8873_typeI        ----------------------------------AKVIRQYSFIFIGITIFLSILTFVIS  201
46170.185_typeI        TSANRLIGAVLMDLHDEWIYSSRKYINFDKMTDEAKVIRQYSFIFIGITIFLSILTFVIS  600
1280.7175_typeI        ----------------------------------AKVIRQYSFIFIGITIFLSILTFVIS  201
1280.7179_typeI        ----------------------------------AKVIRQYSFIFIGITIFLSILTFVIS  201
1280.7178_typeI        ----------------------------------AKVIRQYSFIFIGITIFLSILTFVIS  201
1280.4824_typeI        ----------------------------------AKVIRQYSFIFIGITIFLSILTFVIS  201
1323661.3_typeI        ----------------------------------AKVIRQYSFIFIGITIFLSILTFVIS  201
46170.155_typeI        ----------------------------------AKVIRQYSFIFIGITIFLSILTFVIS  201
1280.3356_typeI        ----------------------------------AKVIRQYSFIFIGITIFLSILTFVIS  201
663951.4_typeI         ----------------------------------AKVIRQYSFIFIGITIFLSILTFVIS  201
1280.7246_typeI        ----------------------------------AKVIRQYSFIFIGITIFLSILTFVIS  201
1280.7245_typeI        ----------------------------------AKVIRQYSFIFIGITIFLSILTFVIS  201
1406863.3_typeI        ----------------------------------AKVIRQYSFIFIGITIFLSILTFVIS  201
1280.3350_typeI        ----------------------------------AKVIRQYSFIFIGITIFLSILTFVIS  201
93062.19_typeI         ----------------------------------AKVIRQYSFIFIGITIFLSILTFVIS  201
1074252.3_typeI        ----------------------------------AKVIRQYSFIFIGITIFLSILTFVIS  201
46170.102_typeI        ----------------------------------AKVIRQYSFIFIGITIFLSILTFVIS  201
1280.3366_typeI        ----------------------------------AKVIRQYSFIFIGITIFLSILTFVIS  201
1280.2220_typeI        ----------------------------------AKVIRQYSFIFIGITIFLSILTFVIS  201
1280.2217_typeI        ----------------------------------AKVIRQYSFIFIGITIFLSILTFVIS  201
1280.2218_typeI        ----------------------------------AKVIRQYSFIFIGITIFLSILTFVIS  201
1280.3574_typeI        ----------------------------------AKVIRQYSFIFIGITIFLSILTFVIS  201
451515.3_typeI         ----------------------------------AKVIRQYSFIFIGITIFLSILTFVIS  201
1458279.3_typeI        ----------------------------------AKVIRQYSFIFIGITIFLSILTFVIS  201
1280.2215_typeI        ----------------------------------AKVIRQYSFIFIGITIFLSILTFVIS  201
46170.148_typeI        ----------------------------------AKVIRQYSFIFIGITIFLSILTFVIS  201
1280.5204_typeI        ----------------------------------AKVIRQYSFIFIGITIFLSILTFVIS  201
1280.4800_typeI        ----------------------------------AKVIRQYSFIFIGITIFLSILTFVIS  201
1280.2216_typeI        ----------------------------------AKVIRQYSFIFIGITIFLSILTFVIS  201
46170.246_typeI        ----------------------------------AKVIRQYSFIFIGITIFLSILTFVIS  201
1280.3352_typeI        ----------------------------------AKVIRQYSFIFIGITIFLSILTFVIS  201
46170.149_typeI        ----------------------------------AKVIRQYSFIFIGITIFLSILTFVIS  201
1280.2219_typeI        ----------------------------------AKVIRQYSFIFIGITIFLSILTFVIS  201
46170.247_typeI        ----------------------------------AKVIRQYSFIFIGITIFLSILTFVIS  201
1280.3566_typeI        ----------------------------------AKVIRQYSFIFIGITIFLSILTFVIS  201
1280.3583_typeI        ----------------------------------AKVIRQYSFIFIGITIFLSILTFVIS  201
451516.9_typeI         ----------------------------------AKVIRQYSFIFIGITIFLSILTFVIS  201
46170.289_typeI        ----------------------------------AKVIRQYSFIFIGITIFLSILTFVIS  201
426430.8_typeI         ----------------------------------AKVIRQYSFIFIGITIFLSILTFVIS  201
1305598.3_typeI        ----------------------------------AKVIRQYSFIFIGITIFLSILTFVIS  201
46170.182_typeI        ----------------------------------AKVIRQYSFIFIGITIFLSILTFVIS  201
46170.290_typeI        ----------------------------------AKVIRQYSFIFIGITIFLSILTFVIS  201
1280.4809_typeI        ----------------------------------AKVIRQYSFIFIGITIFLSILTFVIS  201
1280.3359_typeI        ----------------------------------AKVIRQYSFIFIGITIFLSILTFVIS  201
46170.181_typeI        ----------------------------------AKVIRQYSFIFIGITIFLSILTFVIS  201
46170.183_typeI        ----------------------------------AKVIRQYSFIFIGITIFLSILTFVIS  201
1321369.3_typeI        ----------------------------------AKVIRQYSFIFIGITIFLSILTFVIS  201
1280.10152_typeI       ----------------------------------AKVIRQYSFIFIGITIFLSILTFVIS  201
46170.86_typeI         ----------------------------------AKVIRQYSFIFIGITIFLSILTFVIS  201
1280.4826_typeI        ----------------------------------AKVIRQYSFIFIGITIFLSILTFVIS  201
46170.184_typeI        ----------------------------------AKVIRQYSFIFIGITIFLSILTFVIS  201
1028799.3_typeI        ----------------------------------AKVIRQYSFIFIGITIFLSILTFVIS  201
1280.4849_typeI        ----------------------------------AKVIRQYSFIFIGITIFLSILTFVIS  201
```

```
1280.3589_typeI       -----------------------------------AKVIRQYSFIFIGITIFLSILTFVIS  201
1241616.6_typeI       -----------------------------------AKVIRQYSFIFIGITIFLSILTFVIS  201
546342.4_typeI        -----------------------------------AKVIRQYSFIFIGITIFLSILTFVIS  201
1280.4805_typeI       -----------------------------------AKVIRQYSFIFIGITIFLSILTFVIS  201
93061.5_typeI         -----------------------------------AKVIRQYSFIFIGITIFLSILTFVIS  201


1368166.3_typeI       QFLLKEMKYKRNQEEIETYYEYTLKIEAINNEMRKFRHDYVNILTTLSEYIREDDMPGLR  261
1280.10759_typeI      QFLLKEMKYKRNQEEIETYYEYTLKIEAINNEMRKFRHDYVNILTTLSEYIREDDMPGLR  261
1118959.3_typeI       QFLLKEMKYKRNQEEIETYYEYTLKIEAINNEMRKFRHDYVNILTTLSEYIREDDMPGLR  261
685039.3_typeI        QFLLKEMKYKRNQEEIETYYEYTLKIEAINNEMRKFRHDYVNILTTLSEYIREDDMPGLR  261
1201010.3_typeI       QFLLKEMKYKRNQEEIETYYEYTLKIEAINNEMRKFRHDYVNILTTLSEYIREDDMPGLR  261
1194085.3_typeI       QFLLKEMKYKRNQEEIETYYEYTLKIEAINNEMRKFRHDYVNILTTLSEYIREDDMPGLR  261
1280.3367_typeI       QFLLKEMKYKRNQEEIETYYEYTLKIEAINNEMRKFRHDYVNILTTLSEYIREDDMPGLR  261
1280.5205_typeI       QFLLKEMKYKRNQEEIETYYEYTLKIEAINNEMRKFRHDYVNILTTLSEYIREDDMPGLR  261
523796.5_typeI        QFLLKEMKYKRNQEEIETYYEYTLKIEAINNEMRKFRHDYVNILTTLSEFIREDDMPGLR  261
1280.4850_typeI       QFLLKEMKYKRNQEEIETYYEYTLKIEAINNEMRKFRHDYVNILTTLSEFIREDDMPGLR  261
1280.4852_typeI       QFLLKEMKYKRNQEEIETYYEYTLKIEAINNEMRKFRHDYVNILTTLSEFIREDDMPGLR  261
1155084.3_typeI       QFLLKEMKYKRNQEEIETYYEYTLKIEAINNEMRKFRHDYVNILTTLSEFIREDDMPGLR  261
46170.288_typeI       QFLLKEMKYKRNQEEIETYYEYTLKIEAINNEMRKFRHDYVNILTTLSEFIREDDMPGLR  261
1229492.3_typeI       QFLLKEMKYKRNQEEIETYYEYTLKIEAINNEMRKFRHDYVNILTTLSEFIREDDMPGLR  261
1280.4851_typeI       QFLLKEMKYKRNQEEIETYYEYTLKIEAINNEMRKFRHDYVNILTTLSEFIREDDMPGLR  261
1006543.3_typeI       ------MKYKRNQEEIETYYEYTLKIEAINNEMRKFRHDYVNILTTLSEYIREDDMPGLR  612
46170.245_typeI       QFLLKEMKYKRNQEEIETYYEYTLKIEAINNEMRKFRHDYVNILTTLSEYIREDDMIGLR  261
46170.187_typeI       QFLLKEMKYKRNQEEIETYYEYTLKIEAINNEMRKFRHDYVNILTTLSEYIREDDMIGLR  261
1280.5203_typeI       QFLLKEMKYKRNQEEIETYYEYTLKIEAINNEMRKFRHDYVNILTTLSEYIREDDMIGLR  261
46170.186_typeI       QFLLKEMKYKRNQEEIETYYEYTLKIEAINNEMRKFRHDYVNILTTLSEYIREDDMIGLR  261
1193576.3_typeI       QFLLKEMKYKRNQEEIETYYEYTLKIEAINNEMRKFRHDYVNILTTLSEYIREDDMIGLR  261
1280.8873_typeI       QFLLKEMKYKRNQEEIETYYEYTLKIEAINNEMRKFRHDYVNILTTLSEYIREDDMIGLR  261
46170.185_typeI       QFLLKEMKYKRNQEEIETYYEYTLKIEAINNEMRKFRHDYVNILTTLSEYIREDDMPGLR  660
1280.7175_typeI       QFLLKEMKYKRNQEEIETYYEYTLKIEAINNEMRKFRHDYVNILTTLSEFIREDDMPGLR  261
1280.7179_typeI       QFLLKEMKYKRNQEEIETYYEYTLKIEAINNEMRKFRHDYVNILTTLSEFIREDDMPGLR  261
1280.7178_typeI       QFLLKEMKYKRNQEEIETYYEYTLKIEAINNEMRKFRHDYVNILTTLSEFIREDDMPGLR  261
1280.4824_typeI       QFLLKEMKYKRNQEEIETYYEYTLKIEAINNEMRKFRHDYVNILTTLSEFIREDDMPGLR  261
1323661.3_typeI       QFLLKEMKYKRNQEEIETYYEYTLKIEAINNEMRKFRHDYVNILTTLSEFIREDDMPGLR  261
46170.155_typeI       QFLLKEMKYKRNQEEIETYYEYTLKIEAINNEMRKFRHDYVNILTTLSEFIREDDMPGLR  261
1280.3356_typeI       QFLLKEMKYKRNQEEIETYYEYTLKIEAINNEMRKFRHDYVNILTTLSEYIREDDMPGLR  261
663951.4_typeI        QFLLKEMKYKRNQEEIETYYEYTLKIEAINNEMRKFRHDYVNILTTLSEYIREDDMPGLR  261
1280.7246_typeI       QFLLKEMKYKRNQEEIETYYEYTLKIEAINNEMRKFRHDYVNILTTLSEYIREDDMPGLR  261
1280.7245_typeI       QFLLKEMKYKRNQEEIETYYEYTLKIEAINNEMRKFRHDYVNILTTLSEYIREDDMPGLR  261
1406863.3_typeI       QFLLKEMKYKRNQEEIETYYEYTLKIEAINNEMRKFRHDYVNILTTLSEYIREDDMPGLR  261
1280.3350_typeI       QFLLKEMKYKRNQEEIETYYEYTLKIEAINNEMRKFRHDYVNILTPLSEYIREDDMPGLR  261
93062.19_typeI        QFLLKEMKYKRNQEEIETYYEYTLKIEAINNEMRKFRHDYVNILTPLSEYIREDDMPGLR  261
1074252.3_typeI       QFLLKEMKYKRNQEEIETYYEYTLKIEAINNEMRKFRHDYVNILTTLSEFIREDDMPGLR  261
46170.102_typeI       QFLLKEMKYKRNQEEIETYYEYTLKIEAINNEMRKFRHDYVNILTTLSEFIREDDMPGLR  261
1280.3366_typeI       QFLLKEMKYKRNQEEIETYYEYTLKIEAINNEMRKFRHDYVNILTTLSEFIREDDMPGLR  261
1280.2220_typeI       QFLLKEMKYKRNQEEIETYYEYTLKIEAINNEMRKFRHDYVNILTTLSEYIREDDMPGLR  261
1280.2217_typeI       QFLLKEMKYKRNQEEIETYYEYTLKIEAINNEMRKFRHDYVNILTTLSEYIREDDMPGLR  261
1280.2218_typeI       QFLLKEMKYKRNQEEIETYYEYTLKIEAINNEMRKFRHDYVNILTTLSEYIREDDMPGLR  261
1280.3574_typeI       QFLLKEMKYKRNQEEIETYYEYTLKIEAINNEMRKFRHDYVNILTTLSEYIREDDMPGLR  261
451515.3_typeI        QFLLKEMKYKRNQEEIETYYEYTLKIEAINNEMRKFRHDYVNILTTLSEYIREDDMPGLR  261
1458279.3_typeI       QFLLKEMKYKRNQEEIETYYEYTLKIEAINNEMRKFRHDYVNILTTLSEYIREDDMPGLR  261
1280.2215_typeI       QFLLKEMKYKRNQEEIETYYEYTLKIEAINNEMRKFRHDYVNILTTLSEYIREDDMPGLR  261
46170.148_typeI       QFLLKEMKYKRNQEEIETYYEYTLKIEAINNEMRKFRHDYVNILTTLSEYIREDDMPGLR  261
1280.5204_typeI       QFLLKEMKYKRNQEEIETYYEYTLKIEAINNEMRKFRHDYVNILTTLSEYIREDDMPGLR  261
1280.4800_typeI       QFLLKEMKYKRNQEEIETYYEYTLKIEAINNEMRKFRHDYVNILTTLSEYIREDDMPGLR  261
```

```
1280.2216_typeI        QFLLKEMKYKRNQEEIETYYEYTLKIEAINNEMRKFRHDYVNILTTLSEYIREDDMPGLR  261
46170.246_typeI        QFLLKEMKYKRNQEEIETYYEYTLKIEAINNEMRKFRHDYVNILTTLSEYIREDDMPGLR  261
1280.3352_typeI        QFLLKEMKYKRNQEEIETYYEYTLKIEAINNEMRKFRHDYVNILTTLSEYIREDDMPGLR  261
46170.149_typeI        QFLLKEMKYKRNQEEIETYYEYTLKIEAINNEMRKFRHDYVNILTTLSEYIREDDMPGLR  261
1280.2219_typeI        QFLLKEMKYKRNQEEIETYYEYTLKIEAINNEMRKFRHDYVNILTTLSEYIREDDMPGLR  261
46170.247_typeI        QFLLKEMKYKRNQEEIETYYEYTLKIEAINNEMRKFRHDYVNILTTLSEYIREDDMPGLR  261
1280.3566_typeI        QFLLKEMKYKRNQEEIETYYEYTLKIEAINNEMRKFRHDYVNILTTLSEYIREDDMPGLR  261
1280.3583_typeI        QFLLKEMKYKRNQEEIETYYEYTLKIEAINNEMRKFRHDYVNILTTLSEYIREDDMPGLR  261
451516.9_typeI         QFLLKEMKYKRNQEEIETYYEYTLKIEAINNEMRKFRHDYVNILTTLSEYIREDDMPGLR  261
46170.289_typeI        QFLLKEMKYKRNQEEIETYYEYTLKIEAINNEMRKFRHDYVNILTTLSEYIREDDMPGLR  261
426430.8_typeI         QFLLKEMKYKRNQEEIETYYEYTLKIEAINNEMRKFRHDYVNILTTLSEYIREDDMPGLR  261
1305598.3_typeI        QFLLKEMKYKRNQEEIETYYEYTLKIEAINNEMRKFRHDYVNILTTLSEYIREDDMPGLR  261
46170.182_typeI        QFLLKEMKYKRNQEEIETYYEYTLKIEAINNEMRKFRHDYVNILTTLSEYIREDDMPGLR  261
46170.290_typeI        QFLLKEMKYKRNQEEIETYYEYTLKIEAINNEMRKFRHDYVNILTTLSEYIREDDMPGLR  261
1280.4809_typeI        QFLLKEMKYKRNQEEIETYYEYTLKIEAINNEMRKFRHDYVNILTTLSEYIREDDMPGLR  261
1280.3359_typeI        QFLLKEMKYKRNQEEIETYYEYTLKIEAINNEMRKFRHDYVNILTTLSEYIREDDMPGLR  261
46170.181_typeI        QFLLKEMKYKRNQEEIETYYEYTLKIEAINNEMRKFRHDYVNILTTLSEYIREDDMPGLR  261
46170.183_typeI        QFLLKEMKYKRNQEEIETYYEYTLKIEAINNEMRKFRHDYVNILTTLSEYIREDDMPGLR  261
1321369.3_typeI        QFLLKEMKYKRNQEEIETYYEYTLKIEAINNEMRKFRHDYVNILTTLSEYIREDDMPGLR  261
1280.10152_typeI       QFLLKEMKYKRNQEEIETYYEYTLKIEAINNEMRKFRHDYVNILTTLSEYIREDDMPGLR  261
46170.86_typeI         QFLLKEMKYKRNQEEIETYYEYTLKIEAINNEMRKFRHDYVNILTTLSEYIREDDMPGLR  261
1280.4826_typeI        QFLLKEMKYKRNQEEIETYYEYTLKIEAINNEMRKFRHDYVNILTTLSEYIREDDMPGLR  261
46170.184_typeI        QFLLKEMKYKRNQEEIETYYEYTLKIEAINNEMRKFRHDYVNILTTLSEYIREDDMPGLR  261
1028799.3_typeI        QFLLKEMKYKRNQEEIETYYEYTLKIEAINNEMRKFRHDYVNILTTLSEYIREDDMPGLR  261
1280.4849_typeI        QFLLKEMKYKRNQEEIETYYEYTLKIEAINNEMRKFRHDYVNILTTLSEYIREDDMPGLR  261
1280.3589_typeI        QFLLKEMKYKRNQEEIETYYEYTLKIEAINNEMRKFRHDYVNILTTLSEYIREDDMPGLR  261
1241616.6_typeI        QFLLKEMKYKRNQEEIETYYEYTLKIEAINNEMRKFRHDYVNILTTLSEYIREDDMPGLR  261
546342.4_typeI         QFLLKEMKYKRNQEEIETYYEYTLKIEAINNEMRKFRHDYVNILTTLSEYIREDDMPGLR  261
1280.4805_typeI        QFLLKEMKYKRNQEEIETYYEYTLKIEAINNEMRKFRHDYVNILTTLSEYIREDDMPGLR  261
93061.5_typeI          QFLLKEMKYKRNQEEIETYYEYTLKIEAINNEMRKFRHDYVNILTTLSEYIREDDMPGLR  261
                             *************************************** ***:****** ***

1368166.3_typeI        DYFNKNIVPMKDNLQMNAIKLNGIENLKVREIKGLITAKILRAQEMSIPISIEIPDEVTH  321
1280.10759_typeI       DYFNKNIVPMKDNLQMNAIKLNGIENLKVREIKGLITAKILRAQEMSIPISIEIPDEVTH  321
1118959.3_typeI        DYFNKNIVPMKDNLQMNAIKLNGIENLKVREIKGLITAKILRAQEMSIPISIEIPDEVTH  321
685039.3_typeI         DYFNKNIVPMKDNLQMNAIKLNGIENLKVREIKGLITAKILRAQEMSIPISIEIPDEVTH  321
1201010.3_typeI        DYFNKNIVPMKDNLQMNAIKLNGIENLKVREIKGLITAKILRAQEMSIPISIEIPDEVTH  321
1194085.3_typeI        DYFNKNIVPMKDNLQMNAIKLNGIENLKVREIKGLITAKILRAQEMSIPISIEIPDEVTH  321
1280.3367_typeI        DYFNKNIVPMKDNLQMNAIKLNGIENLKVREIKGLITAKILRAQEMSIPISIEIPDEVTR  321
1280.5205_typeI        DYFNKNIVPMKDNLQMNAIKLNGIENLKVREIKGLITAKILRAQEMSIPISIEIPDEVTR  321
523796.5_typeI         DYFNKNIVPMKDNLQMNAIKLNGIENLKVREIKGLITAKILRAQEMNIPISIEIPDEVTR  321
1280.4850_typeI        DYFNKNIVPMKDNLQMNAIKLNGIENLKVREIKGLITAKILRAQEMNIPISIEIPDEVTR  321
1280.4852_typeI        DYFNKNIVPMKDNLQMNAIKLNGIENLKVREIKGLITAKILRAQEMNIPISIEIPDEVTR  321
1155084.3_typeI        DYFNKNIVPMKDNLQMNAIKLNGIENLKVREIKGLITAKILRAQEMNIPISIEIPDEVTR  321
46170.288_typeI        DYFNKNIVPMKDNLQMNAIKLNGIENLKVREIKGLITAKILRAQEMNIPISIEIPDEVTR  321
1229492.3_typeI        DYFNKNIVPMKDNLQMNAIKLNGIENLKVREIKGLITAKILRAQEMNIPISIEIPDEVTR  321
1280.4851_typeI        DYFNKNIVPMKDNLQMNAIKLNGIENLKVREIKGLITAKILRAQEMNIPISIEIPDEVTR  321
1006543.3_typeI        DYFNKNIVPMKDNLQMNAIKLNGIENLKVREIKGLITAKILRAQEMNIPTSIEIPDEVSS  672
46170.245_typeI        AYFNKNIVPMKDNLQMNAIKLNGIENLKVREIKGLITAKILRAQEMNIPISIEIPDEVSS  321
46170.187_typeI        AYFNKNIVPMKDNLQMNAIKLNGIENLKVREIKGLITAKILRAQEMNIPISIEIPDEVSS  321
1280.5203_typeI        AYFNKNIVPMKDNLQMNAIKLNGIENLKVREIKGLITAKILRAQEMNIPISIEIPDEVSS  321
46170.186_typeI        AYFNKNIVPMKDNLQMNAIKLNGIENLKVREIKGLITAKILRAQEMNIPISIEIPDEVSS  321
1193576.3_typeI        AYFNKNIVPMKDNLQMNAIKLNGIENLKVREIKGLITAKILRAQEMNIPISIEIPDEVSS  321
1280.8873_typeI        AYFNKNIVPMKDNLQMNAIKLNGIENLKVREIKGLITAKILRAQEMNIPISIEIPDEVSS  321
46170.185_typeI        DYFNKNIVPMKDNLQMNAIKLNGIENLKVREIKGLITAKILRAQEMNIPISIEIPDEVSS  720
1280.7175_typeI        DYFNKNIVPMKDNLQMNAIKLNGIENLKVREIKGLITAKILRAQEMNIPISIEIPDEVSS  321
```

```
1280.7179_typeI       DYFNKNIVPMKDNLQMNAIKLNGIENLKVREIKGLITAKILRAQEMNIPISIEIPDEVSS  321
1280.7178_typeI       DYFNKNIVPMKDNLQMNAIKLNGIENLKVREIKGLITAKILRAQEMNIPISIEIPDEVSS  321
1280.4824_typeI       DYFNKNIVPMKDNLQMNAIKLNGIENLKVREIKGLITAKILRAQEMNIPISIEIPDEVSS  321
1323661.3_typeI       DYFNKNIVPMKDNLQMNAIKLNGIENLKVREIKGLITAKILRAQEMNIPISIEIPDEVSS  321
46170.155_typeI       DYFNKNIVPMKDNLQMNAIKLNGIENLKVREIKGLITAKILRAQEMNIPISIEIPDEVSS  321
1280.3356_typeI       DYFNKNIVPMKDNLQMNAIKLNGIENLKVREIKGLITAKILRAQEMNIPISIEIPDEVSS  321
663951.4_typeI        DYFNKNIVPMKDNLQMNAIKLNGIENLKVREIKGLITAKILRAQEMNIPISIEIPDEVSS  321
1280.7246_typeI       DYFNKNIVPMKDNLQMNAIKLNGIENLKVREIKGLITAKILRAQEMNIPISIEIPDEVSS  321
1280.7245_typeI       DYFNKNIVPMKDNLQMNAIKLNGIENLKVREIKGLITAKILRAQEMNIPISIEIPDEVSS  321
1406863.3_typeI       DYFNKNIVPMKDNLQMNAIKLNGIENLKVREIKGLITAKILRAQEMNIPISIEIPDEVSS  321
1280.3350_typeI       DYFNKNIVPMKDNLQMNAIKLNGIENLKVREIKGLITAKILRAQEMNIPISIEIPDEVSS  321
93062.19_typeI        DYFNKNIVPMKDNLQMNAIKLNGIENLKVREIKGLITAKILRAQEMNIPISIEIPDEVSS  321
1074252.3_typeI       DYFNKNIVPMKDNLQMNAIKLNGIENLKVREIKGLITAKILRAQEMNIPISIEIPDEVSS  321
46170.102_typeI       DYFNKNIVPMKDNLQMNAIKLNGIENLKVREIKGLITAKILRAQEMNIPISIEIPDEVSS  321
1280.3366_typeI       DYFNKNIVPMKDNLQMNAIKLNGIENLKVREIKGLITAKILRAQEMNIPISIEIPDEVSS  321
1280.2220_typeI       DYFNKNIVPMKDNLQMNAIKLNGIENLKVREIKGLITAKILRAQEMNFPISIEIPDEVSS  321
1280.2217_typeI       DYFNKNIVPMKDNLQMNAIKLNGIENLKVREIKGLITAKILRAQEMNFPISIEIPDEVSS  321
1280.2218_typeI       DYFNKNIVPMKDNLQMNAIKLNGIENLKVREIKGLITAKILRAQEMNFPISIEIPDEVSS  321
1280.3574_typeI       DYFNKNIVPMKDNLQMNAIKLNGIENLKVREIKGLITAKILRAQEMNFPISIEIPDEVSS  321
451515.3_typeI        DYFNKNIVPMKDNLQMNAIKLNGIENLKVREIKGLITAKILRAQEMNFPISIEIPDEVSS  321
1458279.3_typeI       DYFNKNIVPMKDNLQMNAIKLNGIENLKVREIKGLITAKILRAQEMNFPISIEIPDEVSS  321
1280.2215_typeI       DYFNKNIVPMKDNLQMNAIKLNGIENLKVREIKGLITAKILRAQEMNFPISIEIPDEVSS  321
46170.148_typeI       DYFNKNIVPMKDNLQMNAIKLNGIENLKVREIKGLITAKILRAQEMNFPISIEIPDEVSS  321
1280.5204_typeI       DYFNKNIVPMKDNLQMNAIKLNGIENLKVREIKGLITAKILRAQEMNFPISIEIPDEVSS  321
1280.4800_typeI       DYFNKNIVPMKDNLQMNAIKLNGIENLKVREIKGLITAKILRAQEMNFPISIEIPDEVSS  321
1280.2216_typeI       DYFNKNIVPMKDNLQMNAIKLNGIENLKVREIKGLITAKILRAQEMNFPISIEIPDEVSS  321
46170.246_typeI       DYFNKNIVPMKDNLQMNAIKLNGIENLKVREIKGLITAKILRAQEMNFPISIEIPDEVSS  321
1280.3352_typeI       DYFNKNIVPMKDNLQMNAIKLNGIENLKVREIKGLITAKILRAQEMNFPISIEIPDEVSS  321
46170.149_typeI       DYFNKNIVPMKDNLQMNAIKLNGIENLKVREIKGLITAKILRAQEMNFPISIEIPDEVSS  321
1280.2219_typeI       DYFNKNIVPMKDNLQMNAIKLNGIENLKVREIKGLITAKILRAQEMNFPISIEIPDEVSS  321
46170.247_typeI       DYFNKNIVPMKDNLQMNAIKLNGIENLKVREIKGLITAKILRAQEMNFPISIEIPDEVSS  321
1280.3566_typeI       DYFNKNIVPMKDNLQMNAIKLNGIENLKVREIKGLITAKILRAQEMNFPISIEIPDEVSS  321
1280.3583_typeI       DYFNKNIVPMKDNLQMNAIKLNGIENLKVREIKGLITAKILRAQEMNFPISIEIPDEVSS  321
451516.9_typeI        DYFNKNIVPMKDNLQMNAIKLNGIENLKVREIKGLITAKILRAQEMNFPISIEIPDEVSS  321
46170.289_typeI       DYFNKNIVPMKDNLQMNAIKLNGIENLKVREIKGLITAKILRAQEMNIPISIEIPDEVSS  321
426430.8_typeI        DYFNKNIVPMKDNLQMNAIKLNGIENLKVREIKGLITAKILRAQEMNIPISIEIPDEVSS  321
1305598.3_typeI       DYFNKNIVPMKDNLQMNAIKLNGIENLKVREIKGLITAKILRAQEMNIPISIEIPDEVSS  321
46170.182_typeI       DYFNKNIVPMKDNLQMNAIKLNGIENLKVREIKGLITAKILRAQEMNIPISIEIPDEVSS  321
46170.290_typeI       DYFNKNIVPMKDNLQMNAIKLNGIENLKVREIKGLITAKILRAQEMNIPISIEIPDEVSS  321
1280.4809_typeI       DYFNKNIVPMKDNLQMNAIKLNGIENLKVREIKGLITAKILRAQEMNIPISIEIPDEVSS  321
1280.3359_typeI       DYFNKNIVPMKDNLQMNAIKLNGIENLKVREIKGLITAKILRAQEMNIPISIEIPDEVSS  321
46170.181_typeI       DYFNKNIVPMKDNLQMNAIKLNGIENLKVREIKGLITAKILRAQEMNIPISIEIPDEVSS  321
46170.183_typeI       DYFNKNIVPMKDNLQMNAIKLNGIENLKVREIKGLITAKILRAQEMNIPISIEIPDEVSS  321
1321369.3_typeI       DYFNKNIVPMKDNLQMNAIKLNGIENLKVREIKGLITAKILRAQEMNIPISIEIPDEVSS  321
1280.10152_typeI      DYFNKNIVPMKDNLQMNAIKLNGIENLKVREIKGLITAKILRAQEMNIPISIEIPDEVSS  321
46170.86_typeI        DYFNKNIVPMKDNLQMNAIKLNGIENLKVREIKGLITAKILRAQEMNIPISIEIPDEVSS  321
1280.4826_typeI       DYFNKNIVPMKDNLQMNAIKLNGIENLKVREIKGLITAKILRAQEMNIPISIEIPDEVSS  321
46170.184_typeI       DYFNKNIVPMKDNLQMNAIKLNGIENLKVREIKGLITAKILRAQEMNIPISIEIPDEVSS  321
1028799.3_typeI       DYFNKNIVPMKDNLQMNAIKLNGIENLKVREIKGLITAKILRAQEMNIPISIEIPDEVSS  321
1280.4849_typeI       DYFNKNIVPMKDNLQMNAIKLNGIENLKVREIKGLITAKILRAQEMNIPISIEIPDEVSS  321
1280.3589_typeI       DYFNKNIVPMKDNLQMNAIKLNGIENLKVREIKGLITAKILRAQEMNIPISIEIPDEVSS  321
1241616.6_typeI       DYFNKNIVPMKDNLQMNAIKLNGIENLKVREIKGLITAKILRAQEMNIPISIEIPDEVSS  321
546342.4_typeI        DYFNKNIVPMKDNLQMNAIKLNGIENLKVREIKGLITAKILRAQEMNIPISIEIPDEVSS  321
1280.4805_typeI       DYFNKNIVPMKDNLQMNAIKLNGIENLKVREIKGLITAKILRAQEMNIPISIEIPDEVSS  321
93061.5_typeI         DYFNKNIVPMKDNLQMNAIKLNGIENLKVREIKGLITAKILRAQEMNIPISIEIPDEVSS  321
                       *********************************************.:* ********:
```

1368166.3_typeI	INLNMIDLSRSIGIILDNAIEASTEIDDPIIRVAFIESENSVTFIVMNKCADDIPRIHEL	381
1280.10759_typeI	INLNMIDLSRSIGIILDNAIEASTEIDDPIIRVAFIESENSVTFIVMNKCADDIPRIHEL	381
1118959.3_typeI	INLNMIDLSRSIGIILDNAIEASTEIDDPIIRVAFIESENSVTFIVMNKCADDIPRIHEL	381
685039.3_typeI	INLNMIDLSRSIGIILDNAIEASTEIDDPIIRVAFIESENSVTFIVMNKCADDIPRIHEL	381
1201010.3_typeI	INLNMIDLSRSIGIILDNAIEASTEIDDPIIRVAFIESENSVTFIVMNKCADDIPRIHEL	381
1194085.3_typeI	INLNMIDLSRSIGIILDNAIEASTEIDDPIIRVAFIESENSVTFIVMNKCADDIPRIHEL	381
1280.3367_typeI	INLNMIDLSRSIGIILDNAIEASTEIDDPIIRVAFIESENSVTFIVMNKCADDIPRIHEL	381
1280.5205_typeI	INLNMIDLSRSIGIILDNAIEASTEIDDPIIRVAFIESENSVTFIVMNKCADDIPRIHEL	381
523796.5_typeI	INLNMIDLSRSIGIILDNAIEASSEIDDPIIRVAFIESENSVTFIVMNKCADDIPRIHEL	381
1280.4850_typeI	INLNMIDLSRSIGIILDNAIEASSEIDDPIIRVAFIESENSVTFIVMNKCADDIPRIHEL	381
1280.4852_typeI	INLNMIDLSRSIGIILDNAIEASSEIDDPIIRVAFIESENSVTFIVMNKCADDIPRIHEL	381
1155084.3_typeI	INLNMIDLSRSIGIILDNAIEASSEIDDPIIRVAFIESENSVTFIVMNKCADDIPRIHEL	381
46170.288_typeI	INLNMIDLSRSIGIILDNAIEASSEIDDPIIRVAFIESENSVTFIVMNKCADDIPRIHEL	381
1229492.3_typeI	INLNMIDLSRSIGIILDNAIEASSEIDDPIIRVAFIESENSVTFIVMNKCADDIPRIHEL	381
1280.4851_typeI	INLNMIDLSRSIGIILDNAIEASSEIDDPIIRVAFIESENSVTFIVMNKCADDIPRIHEL	381
1006543.3_typeI	INLNMIDLSRSIGIILDNAIEASTEIDDPIIRVAFIESENSVTFIVMNKCADDIPRIHEL	732
46170.245_typeI	INLNMIDLSRSIGIILDNAIEASTEIDDPIIRVAFIESENSVTFIVMNKCADDIPRIHEL	381
46170.187_typeI	INLNMIDLSRSIGIILDNAIEASTEIDDPIIRVAFIESENSVTFIVMNKCADDIPRIHEL	381
1280.5203_typeI	INLNMIDLSRSIGIILDNAIEASTEIDDPIIRVAFIESENSVTFIVMNKCADDIPRIHEL	381
46170.186_typeI	INLNMIDLSRSIGIILDNAIEASTEIDDPIIRVAFIESENSVTFIVMNKCADDIPRIHEL	381
1193576.3_typeI	INLNMIDLSRSIGIILDNAIEASTEIDDPIIRVAFIESENSVTFIVMNKCADDIPRIHEL	381
1280.8873_typeI	INLNMIDLSRSIGIILDNAIEASTEIDDPIIRVAFIESENSVTFIVMNKCADDIPRIHEL	381
46170.185_typeI	INLNMIDLSRSIGIILDNAIEASTEIDDPIIRVAFIESENSVTFIVMNKCADDIPRIHEL	780
1280.7175_typeI	INLNMIDLSRSIGIILDNAIEASTEIDDPIIRVAFIESENSVTFIVMNKCADDIPRIHEL	381
1280.7179_typeI	INLNMIDLSRSIGIILDNAIEASTEIDDPIIRVAFIESENSVTFIVMNKCADDIPRIHEL	381
1280.7178_typeI	INLNMIDLSRSIGIILDNAIEASTEIDDPIIRVAFIESENSVTFIVMNKCADDIPRIHEL	381
1280.4824_typeI	INLNMIDLSRSIGIILDNAIEASTEIDDPIIRVAFIESENSVTFIVMNKCADDIPRIHEL	381
1323661.3_typeI	INLNMIDLSRSIGIILDNAIEASTEIDDPIIRVAFIESENSVTFIVMNKCADDIPRIHEL	381
46170.155_typeI	INLNMIDLSRSIGIILDNAIEASTEIDDPIIRVAFIESENSVTFIVMNKCADDIPRIHEL	381
1280.3356_typeI	INLNMIDLSRSIGIILDNAIEASTEIDDPIIRVAFIESENSVTFIVMNKCADDIPRIHEL	381
663951.4_typeI	INLNMIDLSRSIGIILDNAIEASTEIDDPIIRVAFIESENSVTFIVMNKCADDIPRIHEL	381
1280.7246_typeI	INLNMIDLSRSIGIILDNAIEASTEIDDPIIRVAFIESENSVTFIVMNKCADDIPRIHEL	381
1280.7245_typeI	INLNMIDLSRSIGIILDNAIEASTEIDDPIIRVAFIESENSVTFIVMNKCADDIPRIHEL	381
1406863.3_typeI	INLNMIDLSRSIGIILDNAIEASTEIDDPIIRVAFIESENSVTFIVMNKCADDIPRIHEL	381
1280.3350_typeI	INLNMIDLSRSIGIILDNAIEASTEIDDPIIRVAFIESENSVTFIVMNKCADDIPRIHEL	381
93062.19_typeI	INLNMIDLSRSIGIILDNAIEASTEIDDPIIRVAFIESENSVTFIVMNKCADDIPRIHEL	381
1074252.3_typeI	INLNMIDLSRSIGIILDNAIEASTEIDDPIIRVAFIESENSVTFIVMNKCADDIPRIHEL	381
46170.102_typeI	INLNMIDLSRSIGIILDNAIEASTEIDDPIIRVAFIESENSVTFIVMNKCADDIPRIHEL	381
1280.3366_typeI	INLNMIDLSRSIGIILDNAIEASTEIDDPIIRVAFIESENSVTFIVMNKCADDIPRIHEL	381
1280.2220_typeI	INLNMIDLSRSIGIILDNAIEASTEIDDPIIRVAFIESENSVTFIVMNKCADDIPRIHEL	381
1280.2217_typeI	INLNMIDLSRSIGIILDNAIEASTEIDDPIIRVAFIESENSVTFIVMNKCADDIPRIHEL	381
1280.2218_typeI	INLNMIDLSRSIGIILDNAIEASTEIDDPIIRVAFIESENSVTFIVMNKCADDIPRIHEL	381
1280.3574_typeI	INLNMIDLSRSIGIILDNAIEASTEIDDPIIRVAFIESENSVTFIVMNKCADDIPRIHEL	381
451515.3_typeI	INLNMIDLSRSIGIILDNAIEASTEIDDPIIRVAFIESENSVTFIVMNKCADDIPRIHEL	381
1458279.3_typeI	INLNMIDLSRSIGIILDNAIEASTEIDDPIIRVAFIESENSVTFIVMNKCADDIPRIHEL	381
1280.2215_typeI	INLNMIDLSRSIGIILDNAIEASTEIDDPIIRVAFIESENSVTFIVMNKCADDIPRIHEL	381
46170.148_typeI	INLNMIDLSRSIGIILDNAIEASTEIDDPIIRVAFIESENSVTFIVMNKCADDIPRIHEL	381
1280.5204_typeI	INLNMIDLSRSIGIILDNAIEASTEIDDPIIRVAFIESENSVTFIVMNKCADDIPRIHEL	381
1280.4800_typeI	INLNMIDLSRSIGIILDNAIEASTEIDDPIIRVAFIESENSVTFIVMNKCADDIPRIHEL	381
1280.2216_typeI	INLNMIDLSRSIGIILDNAIEASTEIDDPIIRVAFIESENSVTFIVMNKCADDIPRIHEL	381
46170.246_typeI	INLNMIDLSRSIGIILDNAIEASTEIDDPIIRVAFIESENSVTFIVMNKCADDIPRIHEL	381
1280.3352_typeI	INLNMIDLSRSIGIILDNAIEASTEIDDPIIRVAFIESENSVTFIVMNKCADDIPRIHEL	381
46170.149_typeI	INLNMIDLSRSIGIILDNAIEASTEIDDPIIRVAFIESENSVTFIVMNKCADDIPRIHEL	381
1280.2219_typeI	INLNMIDLSRSIGIILDNAIEASTEIDDPIIRVAFIESENSVTFIVMNKCADDIPRIHEL	381
46170.247_typeI	INLNMIDLSRSIGIILDNAIEASTEIDDPIIRVAFIESENSVTFIVMNKCADDIPRIHEL	381

```
1280.3566_typeI       INLNMIDLSRSIGIILDNAIEASTEIDDPIIRVAFIESENSVTFIVMNKCADDIPRIHEL  381
1280.3583_typeI       INLNMIDLSRSIGIILDNAIEASTEIDDPIIRVAFIESENSVTFIVMNKCADDIPRIHEL  381
451516.9_typeI        INLNMIDLSRSIGIILDNAIEASTEIDDPIIRVAFIESENSVTFIVMNKCADDIPRIHEL  381
46170.289_typeI       INLNMIDLSRSIGIILDNAIEASTEIDDPIIRVAFIESENSVTFIVMNKCADDIPRIHEL  381
426430.8_typeI        INLNMIDLSRSIGIILDNAIEASTEIDDPIIRVAFIESENSVTFIVMNKCADDIPRIHEL  381
1305598.3_typeI       INLNMIDLSRSIGIILDNAIEASTEIDDPIIRVAFIESENSVTFIVMNKCADDIPRIHEL  381
46170.182_typeI       INLNMIDLSRSIGIILDNAIEASTEIDDPIIRVAFIESENSVTFIVMNKCADDIPRIHEL  381
46170.290_typeI       INLNMIDLSRSIGIILDNAIEASTEIDDPIIRVAFIESENSVTFIVMNKCADDIPRIHEL  381
1280.4809_typeI       INLNMIDLSRSIGIILDNAIEASTEIDDPIIRVAFIESENSVTFIVMNKCADDIPRIHEL  381
1280.3359_typeI       INLNMIDLSRSIGIILDNAIEASTEIDDPIIRVAFIESENSVTFIVMNKCADDIPRIHEL  381
46170.181_typeI       INLNMIDLSRSIGIILDNAIEASTEIDDPIIRVAFIESENSVTFIVMNKCADDIPRIHEL  381
46170.183_typeI       INLNMIDLSRSIGIILDNAIEASTEIDDPIIRVAFIESENSVTFIVMNKCADDIPRIHEL  381
1321369.3_typeI       INLNMIDLSRSIGIILDNAIEASTEIDDPIIRVAFIESENSVTFIVMNKCADDIPRIHEL  381
1280.10152_typeI      INLNMIDLSRSIGIILDNAIEASTEIDDPIIRVAFIESENSVTFIVMNKCADDIPRIHEL  381
46170.86_typeI        INLNMIDLSRSIGIILDNAIEASTEIDDPIIRVAFIESENSVTFIVMNKCADDIPRIHEL  381
1280.4826_typeI       INLNMIDLSRSIGIILDNAIEASTEIDDPIIRVAFIESENSVTFIVMNKCADDIPRIHEL  381
46170.184_typeI       INLNMIDLSRSIGIILDNAIEASTEIDDPIIRVAFIESENSVTFIVMNKCADDIPRIHEL  381
1028799.3_typeI       INLNMIDLSRSIGIILDNAIEASTEIDDPIIRVAFIESENSVTFIVMNKCADDIPRIHEL  381
1280.4849_typeI       INLNMIDLSRSIGIILDNAIEASTEIDDPIIRVAFIESENSVTFIVMNKCADDIPRIHEL  381
1280.3589_typeI       INLNMIDLSRSIGIILDNAIEASTEIDDPIIRVAFIESENSVTFIVMNKCADDIPRIHEL  381
1241616.6_typeI       INLNMIDLSRSIGIILDNAIEASTEIDDPIIRVAFIESENSVTFIVMNKCADDIPRIHEL  381
546342.4_typeI        INLNMIDLSRSIGIILDNAIEASTEIDDPIIRVAFIESENSVTFIVMNKCADDIPRIHEL  381
1280.4805_typeI       INLNMIDLSRSIGIILDNAIEASTEIDDPIIRVAFIESENSVTFIVMNKCADDIPRIHEL  381
93061.5_typeI         INLNMIDLSRSIGIILDNAIEASTEIDDPIIRVAFIESENSVTFIVMNKCADDIPRIHEL  381
                      ***********************:************************************

1368166.3_typeI       FQESFSTKGEGRGLGLSTLKEIADNADNVLLDTIIENGFFIQKVEIINN      430
1280.10759_typeI      FQESFSTKGEGRGLGLSTLKEIADNADNVLLDTIIENGFFIQKVEIINN      430
1118959.3_typeI       FQESFSTKGEGRGLGLSTLKEIADNADNVLLDTIIENGFFIQKVEIINN      430
685039.3_typeI        FQESFSTKGEGRGLGLSTLKEIADNADNVLLDTIIENGFFIQKVEIINN      430
1201010.3_typeI       FQESFSTKGEGRGLGLSTLKEIADNADNVLLDTIIENGFFIQKVEIINN      430
1194085.3_typeI       FQESFSTKGEGRGLGLSTLKEIADNADNVLLDTIIENGFFIQKVEIINN      430
1280.3367_typeI       FQESFSTKGEGRGLGLSTLKEIADNADNVLLDTIIENGFFIQKVEIINN      430
1280.5205_typeI       FQESFSTKGEGRGLGLSTLKEIADNADNVLLDTIIENGFFIQKVEIINN      430
523796.5_typeI        FQESFSTKGEGRGLGLSTLKEIADNADNVLLDTIIENGFFIQKVEIINN      430
1280.4850_typeI       FQESFSTKGEGRGLGLSTLKEIADNADNVLLDTIIENGFFIQKVEIINN      430
1280.4852_typeI       FQESFSTKGEGRGLGLSTLKEIADNADNVLLDTIIENGFFIQKVEIINN      430
1155084.3_typeI       FQESFSTKGEGRGLGLSTLKEIADNADNVLLDTIIENGFFIQKVEIINN      430
46170.288_typeI       FQESFSTKGEGRGLGLSTLKEIADNADNVLLDTIIENGFFIQKVEIINN      430
1229492.3_typeI       FQESFSTKGEGRGLGLSTLKEIADNADNVLLDTIIENGFFIQKVEIINN      430
1280.4851_typeI       FQESFSTKGEGRGLGLSTLKEIADNADNVLLDTIIENGFFIQKVEIINN      430
1006543.3_typeI       FQESFSTKGEGRGLGLSTLKEIADNADNVLLDTIIENGFFIQKVEIINN      781
46170.245_typeI       FQESFSTKGEGRGLGLSTLKEIADNADNVLLDTIIENGFFIQKVEIINN      430
46170.187_typeI       FQESFSTKGEGRGLGLSTLKEIADNADNVLLDTIIENGFFIQKVEIINN      430
1280.5203_typeI       FQESFSTKGEGRGLGLSTLKEIADNADNVLLDTIIENGFFIQKVEIINN      430
46170.186_typeI       FQESFSTKGEGRGLGLSTLKEIADNADNVLLDTIIENGFFIQKVEIINN      430
1193576.3_typeI       FQESFSTKGEGRGLGLSTLKEIADNADNVLLDTIIENGFFIQKVEIINN      430
1280.8873_typeI       FQESFSTKGEGRGLGLSTLKEIADNADNVLLDTIIENGFFIQKVEIINN      430
46170.185_typeI       FQESFSTKGEGRGLGLSTLKEIADNADNVLLDTIIENGFFIQKVEIINN      829
1280.7175_typeI       FQESFSTKGEGRGLGLSTLKEIADNADNVLLDTIIENGFFIQKVEIINN      430
1280.7179_typeI       FQESFSTKGEGRGLGLSTLKEIADNADNVLLDTIIENGFFIQKVEIINN      430
1280.7178_typeI       FQESFSTKGEGRGLGLSTLKEIADNADNVLLDTIIENGFFIQKVEIINN      430
1280.4824_typeI       FQESFSTKGEGRGLGLSTLKEIADNADNVLLDTIIENGFFIQKVEIINN      430
1323661.3_typeI       FQESFSTKGEGRGLGLSTLKEIADNADNVLLDTIIENGFFIQKVEIINN      430
46170.155_typeI       FQESFSTKGEGRGLGLSTLKEIADNADNVLLDTIIENGFFIQKVEIINN      430
1280.3356_typeI       FQESFSTKGEGRGLGLSTLKEIADNADNVLLDTIIENGFFIQKVEIINN      430
```

```
663951.4_typeI          FQESFSTKGEGRGLGLSTLKEIADNADNVLLDTIIENGFFIQKVEIINN      430
1280.7246_typeI         FQESFSTKGEGRGLGLSTLKEIADNADNVLLDTIIENGFFIQKVEIINN      430
1280.7245_typeI         FQESFSTKGEGRGLGLSTLKEIADNADNVLLDTIIENGFFIQKVEIINN      430
1406863.3_typeI         FQESFSTKGEGRGLGLSTLKEIADNADNVLLDTIIENGFFIQKVEIINN      430
1280.3350_typeI         FQESFSTKGEGRGLGLSTLKEIADNADNVLLDTIIENGFFIQKVEIINN      430
93062.19_typeI          FQESFSTKGEGRGLGLSTLKEIADNADNVLLDTIIENGFFIQKVEIINN      430
1074252.3_typeI         FQESFSTKGEGRGLGLSTLKEIADNADNVLLDTIIENGFFIQKVEIINN      430
46170.102_typeI         FQESFSTKGEGRGLGLSTLKEIADNADNVLLDTIIENGFFIQKVEIINN      430
1280.3366_typeI         FQESFSTKGEGRGLGLSTLKEIADNADNVLLDTIIENGFFIQKVEIINN      430
1280.2220_typeI         FQESFSTKGEGRGLGLSTLKEIADNADNVLLDTIIENGFFIQKVEIINN      430
1280.2217_typeI         FQESFSTKGEGRGLGLSTLKEIADNADNVLLDTIIENGFFIQKVEIINN      430
1280.2218_typeI         FQESFSTKGEGRGLGLSTLKEIADNADNVLLDTIIENGFFIQKVEIINN      430
1280.3574_typeI         FQESFSTKGEGRGLGLSTLKEIADNADNVLLDTIIENGFFIQKVEIINN      430
451515.3_typeI          FQESFSTKGEGRGLGLSTLKEIADNADNVLLDTIIENGFFIQKVEIINN      430
1458279.3_typeI         FQESFSTKGEGRGLGLSTLKEIADNADNVLLDTIIENGFFIQKVEIINN      430
1280.2215_typeI         FQESFSTKGEGRGLGLSTLKEIADNADNVLLDTIIENGFFIQKVEIINN      430
46170.148_typeI         FQESFSTKGEGRGLGLSTLKEIADNADNVLLDTIIENGFFIQKVEIINN      430
1280.5204_typeI         FQESFSTKGEGRGLGLSTLKEIADNADNVLLDTIIENGFFIQKVEIINN      430
1280.4800_typeI         FQESFSTKGEGRGLGLSTLKEIADNADNVLLDTIIENGFFIQKVEIINN      430
1280.2216_typeI         FQESFSTKGEGRGLGLSTLKEIADNADNVLLDTIIENGFFIQKVEIINN      430
46170.246_typeI         FQESFSTKGEGRGLGLSTLKEIADNADNVLLDTIIENGFFIQKVEIINN      430
1280.3352_typeI         FQESFSTKGEGRGLGLSTLKEIADNADNVLLDTIIENGFFIQKVEIINN      430
46170.149_typeI         FQESFSTKGEGRGLGLSTLKEIADNADNVLLDTIIENGFFIQKVEIINN      430
1280.2219_typeI         FQESFSTKGEGRGLGLSTLKEIADNADNVLLDTIIENGFFIQKVEIINN      430
46170.247_typeI         FQESFSTKGEGRGLGLSTLKEIADNADNVLLDTIIENGFFIQKVEIINN      430
1280.3566_typeI         FQESFSTKGEGRGLGLSTLKEIADNADNVLLDTIIENGFFIQKVEIINN      430
1280.3583_typeI         FQESFSTKGEGRGLGLSTLKEIADNADNVLLDTIIENGFFIQKVEIINN      430
451516.9_typeI          FQESFSTKGEGRGLGLSTLKEIADNADNVLLDTIIENGFFIQKVEIINN      430
46170.289_typeI         FQESFSTKGEGRGLGLSTLKEIADNADNVLLDTIIENGFFIQKVEIINN      430
426430.8_typeI          FQESFSTKGEGRGLGLSTLKEIADNADNVLLDTIIENGFFIQKVEIINN      430
1305598.3_typeI         FQESFSTKGEGRGLGLSTLKEIADNADNVLLDTIIENGFFIQKVEIINN      430
46170.182_typeI         FQESFSTKGEGRGLGLSTLKEIADNADNVLLDTIIENGFFIQKVEIINN      430
46170.290_typeI         FQESFSTKGEGRGLGLSTLKEIADNADNVLLDTIIENGFFIQKVEIINN      430
1280.4809_typeI         FQESFSTKGEGRGLGLSTLKEIADNADNVLLDTIIENGFFIQKVEIINN      430
1280.3359_typeI         FQESFSTKGEGRGLGLSTLKEIADNADNVLLDTIIENGFFIQKVEIINN      430
46170.181_typeI         FQESFSTKGEGRGLGLSTLKEIADNADNVLLDTIIENGFFIQKVEIINN      430
46170.183_typeI         FQESFSTKGEGRGLGLSTLKEIADNADNVLLDTIIENGFFIQKVEIINN      430
1321369.3_typeI         FQESFSTKGEGRGLGLSTLKEIADNADNVLLDTIIENGFFIQKVEIINN      430
1280.10152_typeI        FQESFSTKGEGRGLGLSTLKEIADNADNVLLDTIIENGFFIQKVEIINN      430
46170.86_typeI          FQESFSTKGEGRGLGLSTLKEIADNADNVLLDTIIENGFFIQKVEIINN      430
1280.4826_typeI         FQESFSTKGEGRGLGLSTLKEIADNADNVLLDTIIENGFFIQKVEIINN      430
46170.184_typeI         FQESFSTKGEGRGLGLSTLKEIADNADNVLLDTIIENGFFIQKVEIINN      430
1028799.3_typeI         FQESFSTKGEGRGLGLSTLKEIADNADNVLLDTIIENGFFIQKVEIINN      430
1280.4849_typeI         FQESFSTKGEGRGLGLSTLKEIADNADNVLLDTIIENGFFIQKVEIINN      430
1280.3589_typeI         FQESFSTKGEGRGLGLSTLKEIADNADNVLLDTIIENGFFIQKVEIINN      430
1241616.6_typeI         FQESFSTKGEGRGLGLSTLKEIADNADNVLLDTIIENGFFIQKVEIINN      430
546342.4_typeI          FQESFSTKGEGRGLGLSTLKEIADNADNVLLDTIIENGFFIQKVEIINN      430
1280.4805_typeI         FQESFSTKGEGRGLGLSTLKEIADNADNVLLDTIIENGFFIQKVEIINN      430
93061.5_typeI           FQESFSTKGEGRGLGLSTLKEIADNADNVLLDTIIENGFFIQKVEIINN      430
                        *************************************************
```

Figure 9: Multiple sequence alignment of canonical type I AgrC proteins and two non-canonical type I AgrC having transposase insertion (*S. aureus* strain T0131 and *S. aureus* strain HC1335) using EMBL-EBI Clustal Omega. Strain IDs are from Patric database and their types are mentioned after the underscore.

The highlighted strains have transposase insertion, and with the alignment it is clear that transposase truncates AgrC (region from residue 175-550) in these two strains which inhibit the gene and subsequently the regulation of *agr* locus.
